# Synthesis, characterization, and performance of the Fe-HAp_Bio_ heterogeneous catalyst for electro-Fenton degradation of cefuroxime sodium

**DOI:** 10.1039/d5ra08148c

**Published:** 2026-01-16

**Authors:** I. Haji, A. Talidi, H. Chakchak, L. Rachidi, A. Zarrouk, G. Kaichouh

**Affiliations:** a Laboratory of Materials, Nanotechnology and Environment, Faculty of Sciences, Mohammed V University in Rabat P. O. Box. 1014 Rabat Morocco g.kaichouh@gmail.com g.kaichouh@um5r.ac.ma +212-672210404; b Laboratory for the Study of Advanced Materials and Application, Faculty of Sciences of Meknes, Higher School of Technology of Meknes, Moulay Ismail University Meknes Morocco; c National Center for Scientific and Technical Research (CNRST-UATRS) Rabat Morocco; d Laboratory of Spectroscopy, Molecular Modeling, Materials, Nanomaterials, Water and Environment (LS3MN2E), CERNE2D, Faculty of Sciences, Mohammed V University in Rabat Morocco azarrouk@gmail.com a.zarrouk@um5r.ac.ma +212-665201397

## Abstract

An efficient heterogeneous Electro-Fenton (EF) process was developed using a catalyst based on hydroxyapatite derived from bovine bone bio-waste (HAp_Bio_), doped with iron through ion exchange (Fe_(*x*)_-HAp_Bio_), for the degradation and mineralization of the antibiotic Cefuroxime Sodium (CFX-Na) in an aqueous medium. The materials were synthesized and characterized by different techniques including X-ray diffraction (XRD), Fourier transform infrared absorption spectroscopy (FTIR), scanning electron microscopy coupled with EDX (SEM-EDX) and X-ray fluorescence spectroscopy (XRF). These analyses demonstrated the high structural stability of HAp_Bio_ despite iron doping, a homogeneous dispersion of iron, the presence of functional groups characteristic of hydroxyapatite such as hydroxyl ions OH^−^, H_2_O, PO_4_^3−^, and CO_3_^2−^, as well as a total iron content of 5.687 wt%. The catalytic activity of the catalyst was evaluated without any prior adjustment to the pH of the solutions. The results showed that an optimal doped iron content of 0.5%, with a catalyst concentration of 1 g L^−1^ applying a current of 400 mA, allowed total degradation to be achieved in 25 min and almost complete mineralization after 5 hours of electrolysis. Radical scavenging experiments using DMSO and chloroform confirmed that hydroxyl radicals (˙OH) were the primary oxidizing species, while hydroperoxyl (˙O_2_H) and superoxide (O_2_˙^−^) radicals were also present in the degradation process. To describe the formation pathways of these reactive species a reaction mechanism was proposed. Also, the catalyst demonstrated good stability after several reuse cycles. Moreover, heterogeneous EF treatment enhanced the biodegradability of the solution after 90 minutes, and therefore, allowed its subsequent low-cost biological treatment. After 17 days, aerobic biological post-treatment achieved almost complete mineralization, which indicated the overall efficiency, sustainability, and less energy consumption of the process.

## Abstract 2

Fe(iii)-doped bio-hydroxyapatite efficiently degrades cefuroxime *via* electro-Fenton, dominated by ˙OH radicals. The catalyst is stable and reusable, and coupling electro-Fenton with aerobic biotreatment achieves near-complete mineralization.

## Graphical abstract

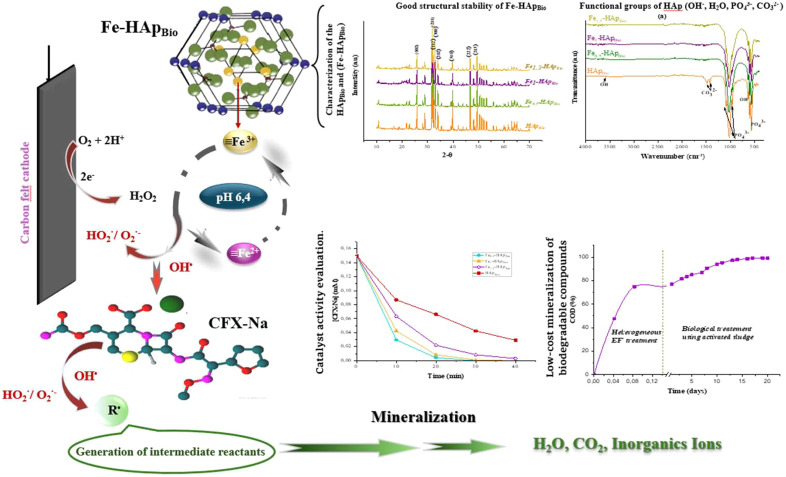

## 1. Introduction

Given the increasingly stringent regulations concerning the protection of natural environments, there is growing interest in the development of new powerful oxidation technologies for the treatment of wastewater contaminated by toxic and non-biodegradable organic compounds. Among these technologies, the electrochemical advanced oxidation process (EAOP) [“Electro-Fenton” (EF) process], based on the Fenton reaction (eqn (1)), has received particular attention as a potentially effective, reliable, and suitable technology for destroying persistent organic micropollutants present in wastewater.^1,2^ This process relies on the *in situ* generation of a large quantity of highly powerful and non-selective reactive species, particularly the hydroxyl radical (˙OH),^3^ which can destroy a wide range of persistent organic contaminants until their total mineralization into CO_2_, H_2_O, and inorganic ions *via* the reaction (eqn (5)), due to its very high oxidation potential (*E*° = 2.8 V/SHE),^4–6^ as well as other oxidizing species such as hydroperoxyl (˙O_2_H) and superoxide (O_2_˙^−^).

In the EF process, dissolved O_2_ is electrochemically reduced to H_2_O_2_ by cathode two-electron reduction, in an acidic solution (eqn (3)),^7,8^ then reacts with ferrous ions to generate highly reactive ˙OH radicals (eqn (1)) through the Fenton reaction (eqn (1)). Furthermore, the ferric ions generated by the Fenton reactions undergo cathodic reduction to generate ferrous ions (eqn (4)), thereby ensuring continuous and efficient catalytic cycle while minimizing the use of chemical and sludge production.^9^ However, the reaction rate decreases due to the consumption of the Fe^2+^ and the formation of Fe^3+^. In fact, by replacing Fe^2+^ with Fe^3+^ or other transition metal ions in the reaction with H_2_O_2_ (eqn (2)), the mechanism proceeds according to a Fenton-like reaction.^10^

1 Fe^2+^ + H_2_O_2_ → Fe^3+^ + OH^−^ + ˙OH (*E*° = 2.81 V)

2 Fe^3+^ + H_2_O_2_ → Fe^2+^ + H^+^ + ˙O_2_H (*E*° = 1.65 V)

3 O_2_ + 2H^+^ + 2e^−^ → H_2_O_2_ (*E*° = 0.69 V/SHE)

4 Fe^3+^ + e^−^ → Fe^2+^ (*E*° = 0.77 V/SHE)

5 Organic pollutants + ˙OH → intermediates + ˙OH → CO_2_ + H_2_O + inorganic ions

Although the homogeneous EF process has a high oxidation capacity, it has a major limitation, including the need for acidic conditions for proper functioning (pH approximately 3), the generation of iron sludge, and the difficulty of recovering the catalyst.^11–13^ In order to overcome these limitations, increasing attention has been given to the development of heterogeneous EF systems,^14^ using poorly soluble or insoluble solid catalysts in water.^12^

These systems offer several advantages that include an extended pH operating range, high efficiency in H_2_O_2_ decomposition, high stability, and the reusability of the catalyst that reduces costs and resources consumption, and the ability to produce radicals both on the catalyst surface and in solution.^15,16^

In this context, impregnating or immobilizing iron species onto solid porous supports has proven to be an effective solution, avoiding the use of dissolved iron salts, minimizing iron leaching, and ensuring more sustainable catalysts use.^17^ Therefore, the selection of an effective support material is important in designing a high-performance heterogenous catalyst. The support choice mainly depends on its nature and proprieties.^18^ In fact, an ideal support material should: (i) be chemically inert while promoting strong physicochemical interactions with iron species at the support without altering their properties an reactivity, (ii) high specific surface area, (iii) good adsorption capacity toward both iron and target organic pollutants (iv) physical structure that allows liquid and solid phase separation, and (v) simple reactor design that allows the occurrence of mass transfer processes.^19^

As such, several porous materials with large surface areas have been investigated as potential supports, including clays,^20^ zeolites,^21^ activated carbon,^22^ biochar,^23^ activated carbon fiber, and nanofiber. Among these materials, hydroxyapatite (HAp), with the chemical formula Ca_10_(PO_4_)_6_(OH)_2_, has proven to be an effective catalyst support that can meet the above-mentioned requirements due to its interesting and unique physicochemical properties, such as thermal and mechanical stability, ion-exchange capacity, biocompatibility, and excellent metal ion adsorption capacity.^24^ It also exhibits very low water solubility and high stability during oxidation.^25^ Although widely studied in the medical field, HAp is also attracting growing interest as an adsorbent for treating water or soil contaminated with heavy metals and radioactive waste, due to its porosity and excellent adsorption capacity^26,27^ and as a catalyst^28–30^ and photocatalyst.^31^

Generally, Hap is synthesized from calcium- and phosphorus-containing compounds by various methods, including co-precipitation, sol–gel, and hydrothermal methods.^32^ However, these synthesis processes can be complex and require the use of expensive chemical reagents or strict operating conditions. For this reason, the green synthesis of hydroxyapatite from bio-waste sources such as mammalian bones including those of bovines, ostriches, and chickens; as well as fish bones and scales, shells, plants, algae, and even mineral sources, has been developed as a clean, cost-effective, biocompatible, environmentally friendly, and above all, non-toxic method.^33^ Indeed, transforming this waste into value-added materials will significantly enhance sustainable economic development and pave the way for more efficient waste management.

Among the sources mentioned, bovine bones represent the most established source for HAp extraction due to their ease of use, low cost, abundance, and especially their high thermal stability.^34^ Based on dry weight, these bones are composed of 65–70% hydroxyapatite and 30–35% organic matter.^35^ Moreover, natural HAp derived from bones contains various inorganic elements such as phosphorus (P), calcium (Ca), magnesium (Mg), boron (B), iron (Fe), manganese (Mn), potassium (K), copper (Cu), and zinc (Zn),^36,37^ which give it interesting structural and catalytic properties, making it valuable in several applications.

These promising characteristics of biologically sourced HAp prompted us to prepare a catalyst based on HAp extracted from bovine bones, doped with iron by ion exchange, and to evaluate its performance for the first time in the heterogeneous electro-Fenton process for the degradation and mineralization in aqueous medium of the antibiotic Cefuroxime Sodium (CFX-Na).

## 2. Experimental section

### 2.1. Chemicals and materials

Cefuroxime Sodium (CFX-Na, C_16_H_15_N_4_NaO_8_S) was supplied by MedChemExpress. Iron nitrate (Fe(NO_3_)_3_·9H_2_O, 99%), sodium sulfate (Na_2_SO_4_, >99% (support electrolyte)), sulfuric acid (H_2_SO_4_, 96%, used for pH adjustment), and potassium chloride (KCl) were purchased from Shanghai Chemicals (Shanghai, China). Dimethyl sulfoxide (DMSO, C_2_H_6_OS, 99.7%) and chloroform (CHCl_3_, ≥99%) were obtained from Sigma-Aldrich. All chemical reagents, including the CFX-Na antibiotic, were of analytical grade and used directly without any additional purification. Aqueous solutions were prepared using double-distilled water obtained using a double distiller L-4B system.

### 2.2. Preparation of Fe_(*x*)_-HAp_Bio_ catalyst

#### 2.2.1. Preparation of hydroxyapatite support from biowaste (HAp_Bio_)

**Fig. 1:**
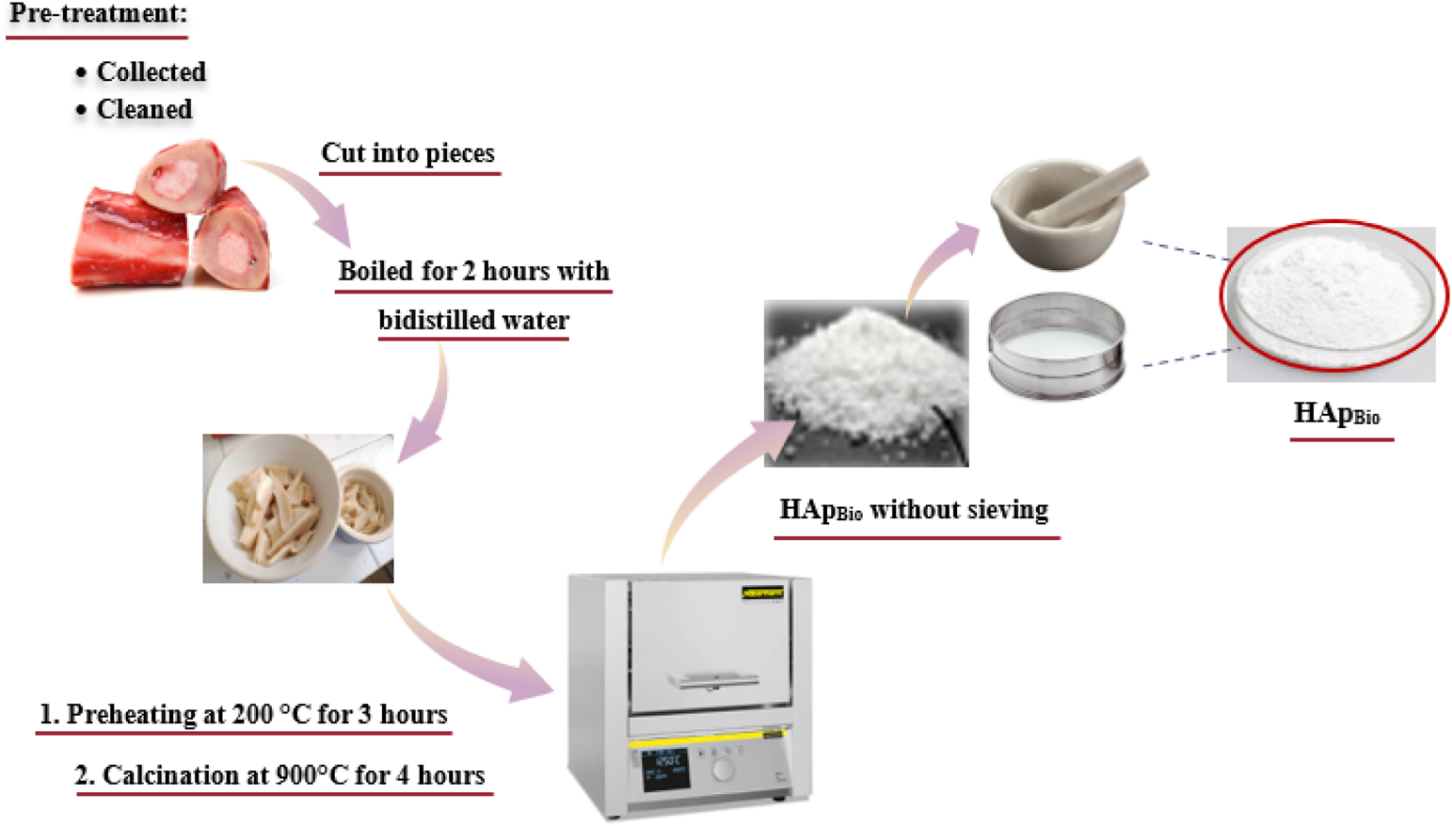
Synthesis process of bio-hydroxyapatite (HAp_Bio_).

Natural hydroxyapatite (HAp_Bio_) was extracted by calcining bovine bones collected from a local butcher in the Rabat/Sale region, Morocco (Fig. 1).

After collection, the bovine bones were manually cleaned to remove residual meat, fat, and outer membrane. They were then cut into small pieces using a cutter and boiled for 2 hours in double-distilled water to remove any remaining lipids and impurities. Following several rinses with double-distilled water, the bones underwent a two main step thermal treatment:

• Preheating at 200 °C for 3 hours to remove organic matter and internal fat;

• Calcination at 900 °C for 4 hours at a heating rate of 10 °C min^−1^ in an electric muffle furnace (Nabertherm).

The resulting powder was slowly cooled in the furnace to ensure the formation of the apatite phase. The obtained biomaterial (HAp_Bio_) was sieved to achieve a particle size of approximately 125 µm.

#### 2.2.2. Preparation of the Fe_(*x*)_-HAp_Bio_ catalyst

**Fig. 2:**
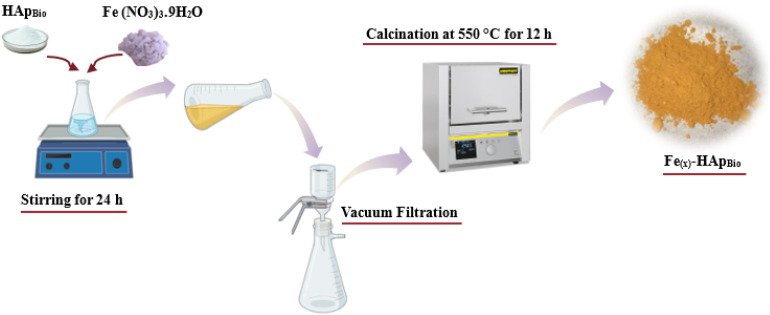
Synthesis process of the Fe_(*x*)_-HAp_Bio_ catalyst.

The Fe_(*x*)_-HAp_Bio_ catalyst was prepared by impregnating iron(iii) using an iron nitrate solution through the ion exchange method. The impregnation process (Fig. 2) involved incorporating various amounts of iron (0.5, 1, and 1.5 wt%) into 1 g of the hydroxyapatite bio-support (HAp_Bio_), used as a catalyst carrier. The iron precursor solution was prepared by dissolving iron nitrate nonahydrate (Fe(NO_3_)_3_·9H_2_O) in 100 mL of double-distilled water. The resulting suspension was stirred at room temperature for 24 hours.

The solid obtained after filtration was washed with hot water, dried at 80 °C, and then calcined at 550 °C for 12 hours with a heating rate of 10 °C min^−1^. The resulting catalytic materials are designated Fe_(*x*)_-HAp_Bio_, where *x* indicates the weight percentage of the active iron(iii) phase.

### 2.3. Characterization of Fe_(*x*)_-HAp_Bio_ catalyst

Catalysts crystallinity was evaluated by X-ray diffraction (XRD) using a Bruker D8 Advance diffractometer equipped with a monochromatic copper (CuKα) radiation source with a wavelength of *λ* = 1.5406 Å, operating at 40 kV and 250 Ma. The analyses were performed in a 2*θ* range of 10–80°, with a scanning speed of 1° min^−1^.

The functional groups present in pure biological hydroxyapatite (HAp_Bio_) and iron-doped hydroxyapatite (Fe_(*x*)_-HAp_Bio_) were identified using Fourier transform infrared absorption spectroscopy (FTIR). The spectra were recorded at room temperature on a Bruker Equinox 55 spectrometer, in transmission mode, in a spectral range of 4000 to 400 cm^−1^ using KBr pellets.

Materials morphology was examined under a controlled atmosphere using a scanning electron microscope (SEM) (Quattro ESEM) coupled with an EDS microanalyzer (resolution of 129 eV).

A semi-quantitative multi-element analysis of the Fe_0.5_-HAp_Bio_ material was performed by X-ray fluorescence spectroscopy (XRF) at room temperature using a wavelength dispersive spectrometer (WDS), measuring elements ranging from Be to U.

### 2.4. Experimental procedure

#### 2.4.1. Heterogeneous electrochemical reactor

Heterogeneous phase electrochemical degradation and mineralization tests of CFX-Na were performed in a 200 mL electrolytic cell with three electrodes. A platinum (Pt) anode (2.5 cm × 2 cm), a carbon felt cathode (6 cm × 5 cm × 0.5 cm), and a saturated KCl calomel reference electrode was used. The system was powered by direct current from a PGZ301 (Voltalab) potentiostat/galvanostat operating in galvanostatic mode.

An initial concentration of 0.15 mM CFX Na was introduced into the cell, with a predefined amount of Fe_(*x*)_-HAp_Bio_ catalyst, adding 0.05 M Na_2_SO_4_ (support electrolyte). Compressed air was continuously introduced to ensure the saturation of the solution with O_2_ necessary for the electro-generation of H_2_O_2_ in the cathode (eqn (3)). All experiments were carried out at room temperature, and samples were taken at regular intervals, filtered, and analyzed.

#### 2.4.2. Aerobic biological reactor

Duplicate cultures were carried out in Erlenmeyer flasks containing 200 mL of a pre-electrolyzed CFX-Na solution (0.15 mM), treated *via* the heterogeneous electro-Fenton process. The flasks were sealed with cotton plug to allow proper oxygenation. Minerals and trace elements were added to the culture medium, and the pH was adjusted to 7 using NaOH.^38^ Activated sludge from a local wastewater treatment plant (Ain El Aouda) was added at an initial concentration of 1 g L^−1^ of dry matter. The cultures were stirred at room temperature, and 5 mL samples were taken periodically, filtered, and injected for COD analysis.

### 2.5. Analytical methods

CFX-Na concentration was monitored by high-performance liquid chromatography (HPLC) using a DIONEX UltiMate 3000 system, according to the previously described protocol.^38^ Dissolved iron ion concentration was measured by inductively coupled plasma optical emission spectrometry (ICP-OES) using an Ultima Jobin Yvon system. CFX-Na adsorption was monitored using a UV-vis spectrophotometry (Jasco V-730, Japan) at the maximum absorption wavelength of 273 nm.

The point of zero charge (pH_zpc_) of the Fe_0.5_-HAp_Bio_ catalyst was determined by preparing a series of NaNO_3_ (0.1 M) solutions. The pH of each solution was adjusted between 3 and 12 by adding HCl or NaOH using a pH meter (Thermo Fisher Scientific). Then, 0.1 g of catalyst was added to 25 mL of each solution. The mixtures were stirred for 24 hours at room temperature, then filtered before measuring the final pH. The pH_pzc_ value was deduced from the intersection point of the ΔpH curve (ΔpH = pH_f_ − pH_i_) with the *X*-axis and the pH_i_ value.^39^

Solutions mineralization was assessed by measuring the Chemical Oxygen Demand (COD) using the dichromate method and a DR/125 spectrophotometer (Hach, USA). The biodegradability of the treated and untreated solutions was evaluated by measuring the Biological Oxygen Demand (BOD_5_) using an OxiDirect system, following the respirometric evaluation method over 5 days at 20 °C in the dark.

The apparent rate constants (*K*_app_) of the pseudo-first-order model were determined according to eqn (6), where *C*_0_ and *C*_*t*_ are the pollutant concentrations at initial time and at time *t*, respectively, and *t* is the electrolysis time (min).6
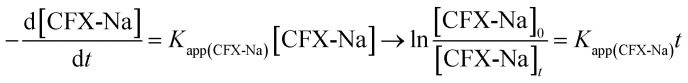


The energy consumption (EC, in kWh g^−1^) was calculated using eqn (7), where *E*_cell_ is the average cell voltage (V), *I* is the applied current (A), (ΔDCO)_*t*_ is the change in COD (g L^−1^), *V* is the volume of the solution (L), and *t* is the electrolysis time (h).7
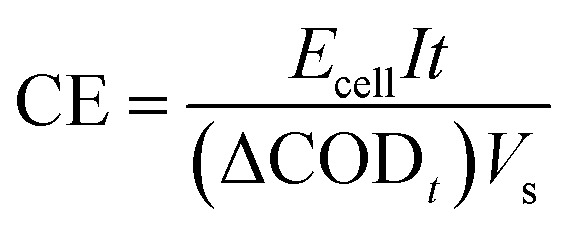


## 3. Results and discussion

### 3.1. Characterization of the HAp_Bio_ support and the (Fe_(*x*)_-HAp_Bio_) catalyst

#### 3.1.1. Structural analysis by XRD

**Fig. 3:**
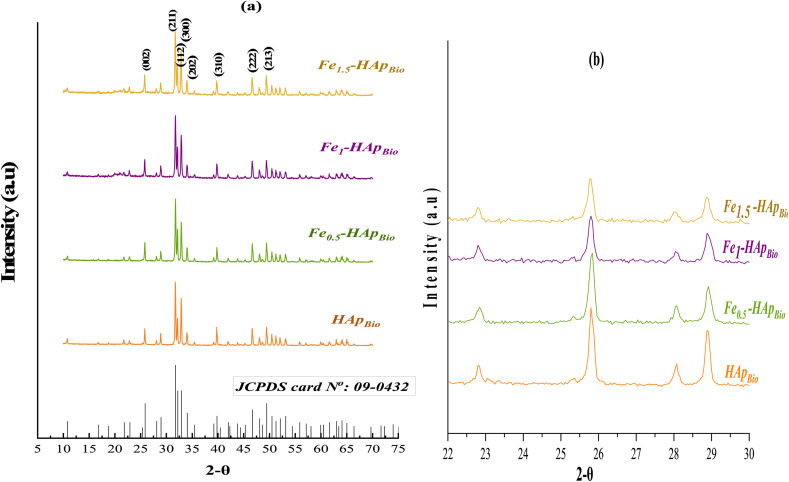
XRD diffractograms of HAp_Bio_ and Fe_(*x*)_-HAp_Bio_: (a) overall view, (b) zoomed views.

X-ray diffraction analysis of the hydroxyapatite support particles, both pure (HAp_Bio_) and iron-doped at various loadings (Fe_(*x*)_-HAp_Bio_) (Fig. 3) was performed to confirm the formation and structural purity of hydroxyapatite and to evaluate the impact of iron incorporation on its crystalline structure.

The diffractogram of the pure HAp_Bio_ (Fig. 3(a)) exhibits exactly the same characteristic diffractogram of the crystalline structure of hydroxyapatite, according to the JCPDS card no.: 09-0432, corresponding to the hexagonal system with space group *P*6_3_/*m*, with no secondary phases detected, confirming a well-crystallized structure. The main diffraction peaks of HAp were observed at 2*θ* values around: 25.9°, 31.8°, 32.2°, 32.9°, 34.1°, 39.9°, 46.7°, and 49.5°, corresponding to the (002), (211), (112), (300), (202), (310), (222) and (213) crystal planes, respectively.

After iron doping (Fe_(*x*=0.5,1,1.5)_-HAp_Bio_), the characteristic peaks of HAp_Bio_ remain present, indicating that the crystalline structure is retained despite the incorporation of iron. Furthermore, the absence of additional peaks confirms the lack of secondary phases such as iron oxides, indicating that ion exchange with iron does not affect the crystalline structure of hydroxyapatite.

However, a slight shift of the peaks towards higher 2*θ* angles was observed, particularly in the zoomed part of the diffractograms (Fig. 3(b)). This shift was accompanied by a slight decrease in peak intensity and a broadening of certain peaks, which became more pronounced at higher iron contents. These changes can be attributed to a decrease in crystallinity due to the partial substitution of calcium ions Ca^2+^ (0.99 Å) by iron ions (0.66 Å), which inhibits the crystal growth of hydroxyapatite.^40–42^

#### 3.1.2. FTIR spectroscopic analysis

**Fig. 4:**
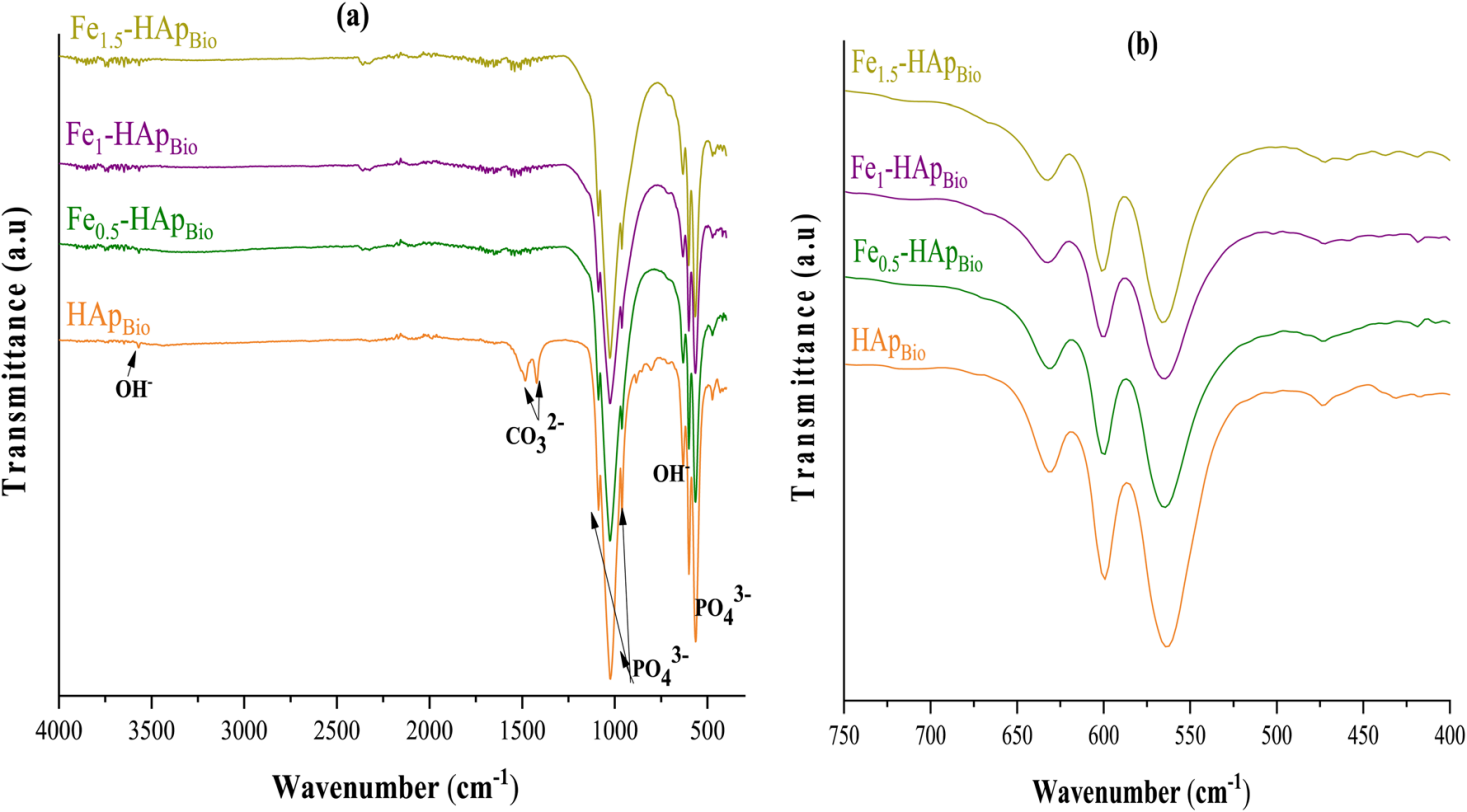
FTIR spectra: (a) overall spectra of undoped bio-hydroxyapatite particles (HAp_Bio_) and iron(iii)-doped ones (Fe_(*x*)_-HAp_Bio_), (b) zoomed-in region from 750–400 cm^−1^.

The FTIR spectra of pure HAp_Bio_ and iron-doped at different percentages (Fe_(*x*=0.5,1,1.5)_-HAp_Bio_) (Fig. 4) allowed the identification of characteristic functional groups.

The bands corresponding to the stretching vibration of hydroxyl groups (OH^−^) are clearly observed at 3568 and 631 cm^−1^.^43^ The region between 1000 and 1100 cm^−1^ corresponds to the characteristic phosphate (PO_4_^3−^) bands, an essential component of the HAp structure. More specifically, the bands observed around 1035, 1043, and 1093 cm^−1^ are attributed to the antisymmetric stretching mode (*ν*_3_) of PO_4_^3−^.

In the lower frequency region, the peaks at 475 and 963 cm^−1^ are assigned to the symmetric stretching modes (*ν*_2_ and *ν*_1_, respectively), while the bands at 567 and 604 cm^−1^ are attributed to the bending vibration mode (*ν*_4_) of PO_4_^3−^.^44^ These bands are very typical of hydroxyapatite, and confirm the integrity of phosphate group in crystalline lattice. Moreover, the presence of carbonate groups (CO_2_^3−^), typical of biological hydroxyapatites, is also used confirmed by the adsorption bands between 1460 cm^−1^ and 1530 cm^−1^.^45^ Iron doping causes considerable variation in the intensity and width of these bands, in particular in the phosphate and hydroxyl groups.

Moreover, the absence of other bands indicates that iron is successfully incorporated in the HAp crystal structure without any formation of secondary phases, such as iron oxides.

#### 3.1.3. SEM-EDX morphological analysis

**Fig. 5:**
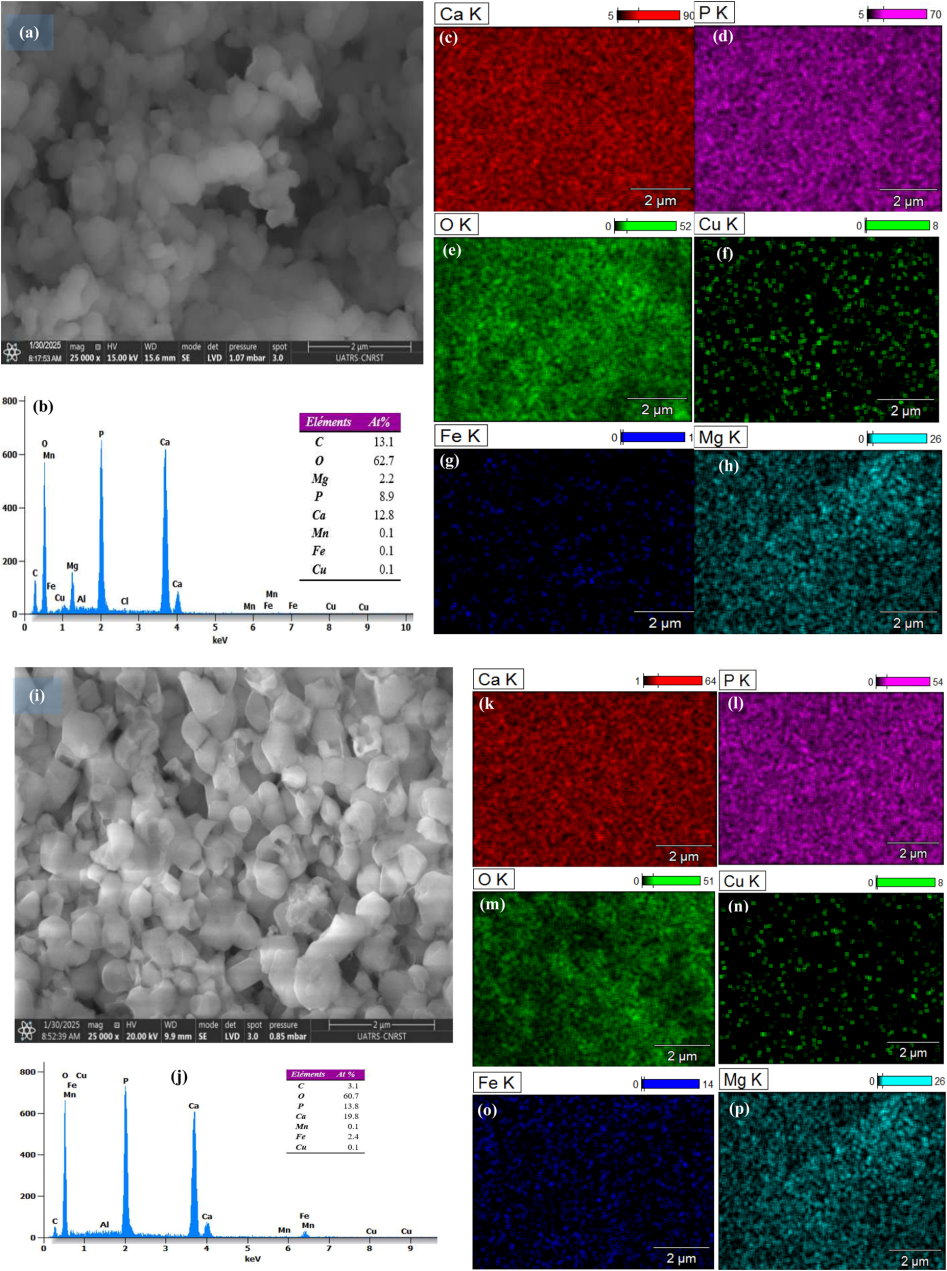
SEM images of: (a) HAp_Bio_; (i) Fe_0.5_-HAp_Bio_; (b and j) EDX spectra, and elemental distribution maps for Ca (c and k), P (d and l), O (e and m), Cu (f and n), Fe (g and o), and Mg (h and p).

The SEM images analysis (Fig. 5(a and i)) shows that, after iron incorporation into the HAp_Bio_ matrix (Fig. 5(i)), the particles initially characterized by a granular structure with mostly circular/spherical shapes evolved into a more granular morphology, displaying variable particle sizes and angular edges. This observation implies the agglomeration of grains having different sizes. In addition, the EDX spectra obtained (Fig. 5(b and j)) confirmed that the material is composed of calcium Ca, phosphorus P, and oxygen O. The traces of other inorganic elements, such as Fe, Cu, Mn, or Mg, naturally present in the bone, are also verified.

Furthermore, the appearance of additional Fe peaks in iron-doped sample (Fe_0.5_-HAp_Bio_) (Fig. 5(g)) confirms the successful incorporation of iron into the material structure.

Elemental mapping (Fig. 5(c, k, d, l, e, m, f, n, g, o, h and p)) shows a homogeneous distribution of calcium, phosphorus, and oxygen, indicating the uniformity of the hydroxyapatite phase. However, trace elements and doped elements, such as iron and copper, appear as dispersed spots, suggesting a localized presence within the matrix.

The elemental composition of the Fe_0.5_-HAp_Bio_ catalyst was determined by X-ray fluorescence spectroscopy (XRF) and demonstrated the presence of 5.687 wt% Fe, confirming the effective impregnation of iron into the biological hydroxyapatite matrix (Table 1). The analysis also revealed the predominant presence of Ca, O, and P, characteristic elements of the typical hydroxyapatite structure. In addition, trace elements such as Mg and Cu were also detected, which probably originate from the bone support used.

**Table 1:** Elemental composition of the Fe_0.5_-HAp_Bio_ catalyst

Element	Weight (%)
Ca	28.48
O	43.25
P	20.78
Mg	0.1075
Fe	5.687
Cu	0.008961

### 3.2. Performance of the biocatalyst in the aqueous degradation and mineralization of CFX-Na

#### 3.2.1. Catalytic behavior of Fe_(*x*)_-HAp_Bio_ at different iron contents

**Fig. 6:**
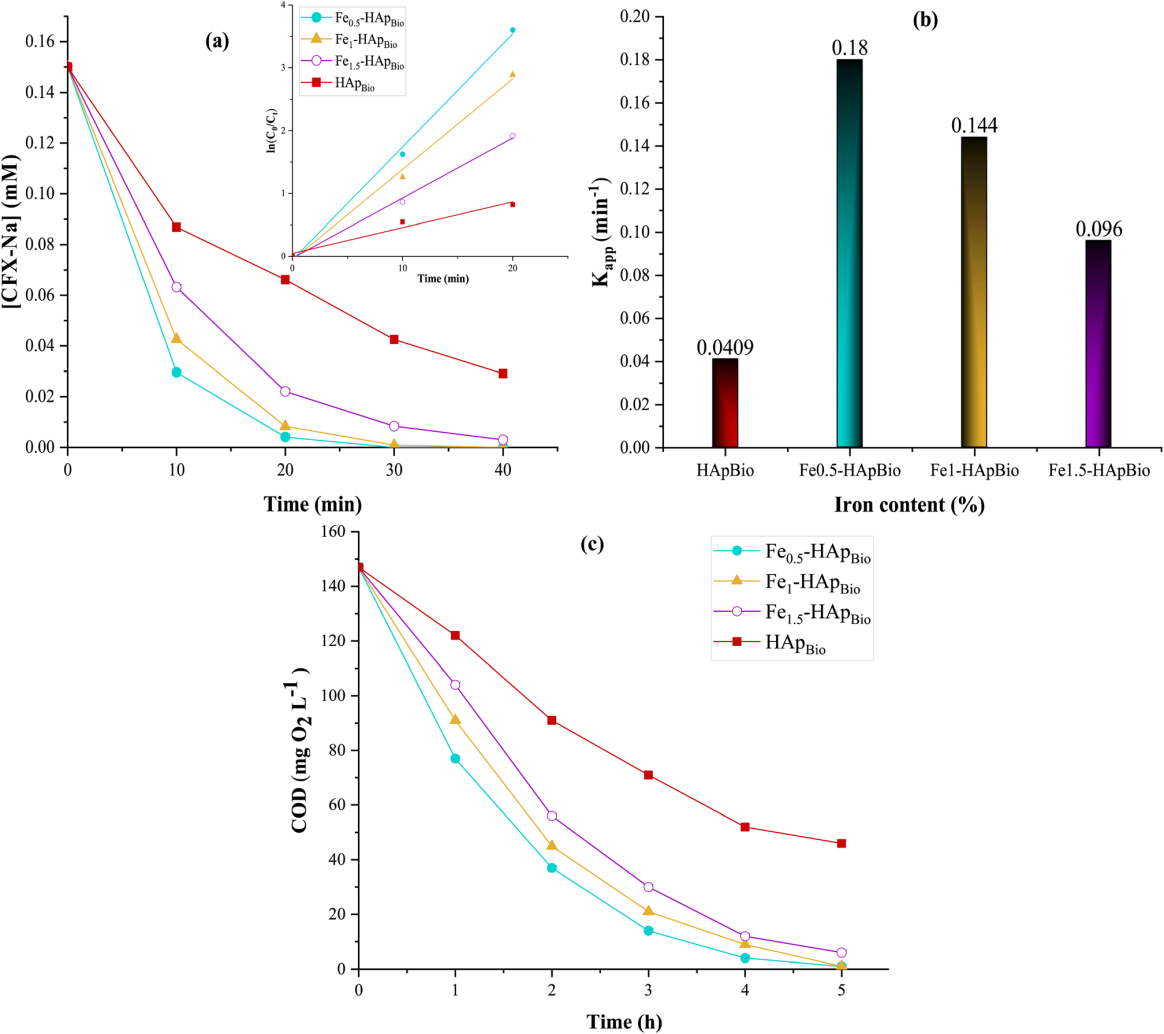
Effect of iron content supported on HAp_Bio_ surface on: (a) degradation kinetics, (b) apparent rate constants (*K*_app_), and (c) COD evolution, during heterogeneous EF treatment of CFX-Na: [CFX-Na]_0_ = 0.15 mM, *I* = 400 mA, [Fe_(*x*)_-HAp_Bio_]_0_ = 1 g L^−1^, initial solution pH = 6.4.

In order to evaluate the contribution of the HAp_Bio_ structure and the content of incorporated iron to the degradation and mineralization of CFX-Na, experiments were carried out using pure HAp_Bio_ and iron-doped simples at various percentages (Fe_(*x*)_-HAp_Bio_, *x* = 0.5, 1 and 1.5%). Fig. 6 illustrates the evolution of CFX-Na concentrations and COD during electrolysis at an initial solution pH of 6.4.

In all cases, as shown in Fig. 6(a), the electrochemical oxidation of CFX-Na was significantly more pronounced in the presence of iron-doped HAp_Bio_ compared to pure HAp_Bio_, highlighting the catalytic role of the incorporated iron species, which promote the generation of oxidizing species responsible for the degradation of the organic pollutant. Indeed, after 25 minutes of electrolysis, complete removal of CFX-Na was achieved using Fe_0.5_-HAp_Bio_. This confirms that at this iron content (0.5%), the catalyst provides more active catalytic sites, enhancing the catalytic reaction with H_2_O_2_ and thus leading to a higher production of reactive species, which improves the heterogeneous degradation rate of CFX-Na.

However, as the iron content increased from 0.5 to 1.5%, the CFX-Na removal rate decreased from 98 to 85.26% after 20 min of electrolysis, and the apparent rate constant dropped from 0.18 to 0.096 min^−1^ (Fig. 6(b)). A similar trend was observed during the mineralization of the solution (Fig. 6(c)); the mineralization efficiency decreased from 98% for 0.5% Fe to 91% for 1.5% Fe after 4 hours of treatment. This could be attributed to a reduction in the number of active surface sites with higher iron loadings, as well as the promotion of competitive side reactions between the oxidizing radicals and excess iron ions, which consume reactive species and thus reduce the degradation performance of the targeted antibiotic.

On the other hand, the bio-support HAp_Bio_ alone also catalysed the oxidation of CFX-Na, but at a lower rate than with iron doped catalysts, with a degradation rate of approximately 71.7% after 30 minutes of treatment. This activity may be due, on the one hand, in part, to the presence of trace elements such as Fe in the bone derived hydroxyapatite, as previously noted.^46^

Although present in low concentration, these trace elements may partially contribute to redox reactions responsible for CFX-Na degradation. Additionally, this activity could also be related to the hydroxyl OH^−^ groups present in the hydroxyapatite structure, which, in the presence of H_2_O_2_, may generate oxidizing radicals in a manner similar to metal-catalyzed homogeneous Fenton reactions.^47^

#### 3.2.2. Properties and behavior of the Fe_0.5_-HAp_Bio_ catalyst in aqueous solution

Before optimizing the parameters influencing the oxidation of CFX-Na by the EF process using Fe_0.5_-HAp_Bio_ as a heterogeneous catalyst, preliminary experiments were conducted to evaluate, on the one hand, the adsorption properties of the molecule on the material, and on the other hand, the behavior of the aqueous solution in the presence of the catalyst, such as the generation of H^+^ and Fe^2+^/Fe^3+^ ions during treatment.

##### 3.2.2.1. Adsorption of CFX-Na onto Fe_0.5_-HAp_Bio_

To better understand the interaction mechanisms between CFX-Na and both pure HAp_Bio_ and iron-doped HAp_Bio_ (Fe_0.5_-HAp_Bio_), adsorption tests were carried out by bringing the materials into contact with an aqueous solution of CFX-Na (0.15 mM) without any applied electric field.

**Fig. 7:**
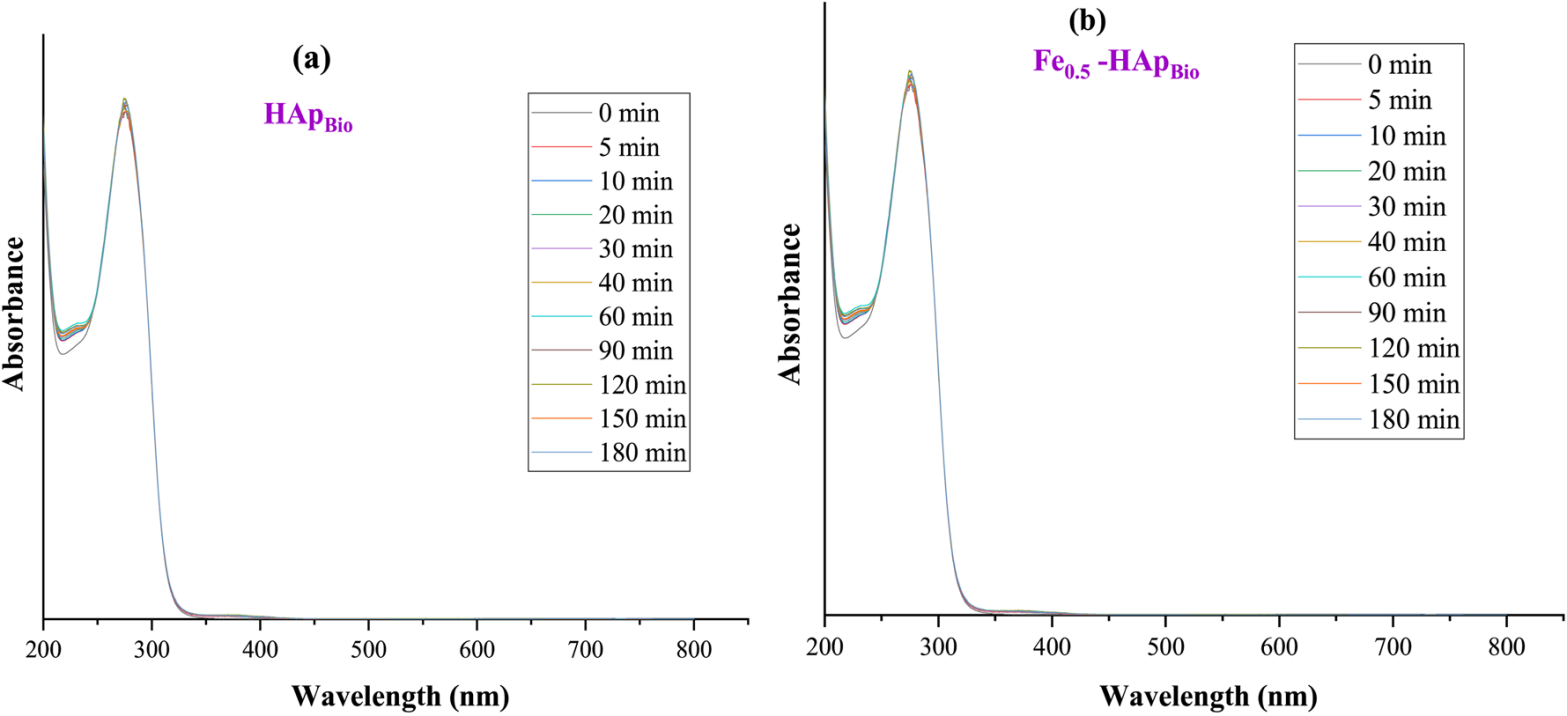
Evaluation of the adsorbent properties of hydroxyapatite: (a) pure (HAp_Bio_), (b) iron-doped (Fe_0.5_-HAp_Bio_).

These tests were conducted to assess their capacity to adsorb CX-Na, a phenomenon that could potentially act as a competitor to the electrochemical oxidation during the EF process and therefore affect the overall efficiency of the treatment process. CFX-Na concentration was monitored using a UV-visible spectrophotometer at a wavelength of 237 nm. The results obtained are presented as a function of contact time in Fig. 7.

According to the figure, no significant variation in CFX-Na absorbance was observed, which could indicate limited or even negligible adsorption of the pollutant onto the support material, whether pure HAp_Bio_ (Fig. 7(a)) or iron-doped Fe_0.5_-HAp_Bio_ (Fig. 7(b)).

In order to better understand this behavior, the point of zero charge of the Fe_0.5_-HAp_Bio_ catalyst was determined. The pH_pzc_ corresponds to the pH at which the surface of the material has a net zero charge.^48^

**Fig. 8:**
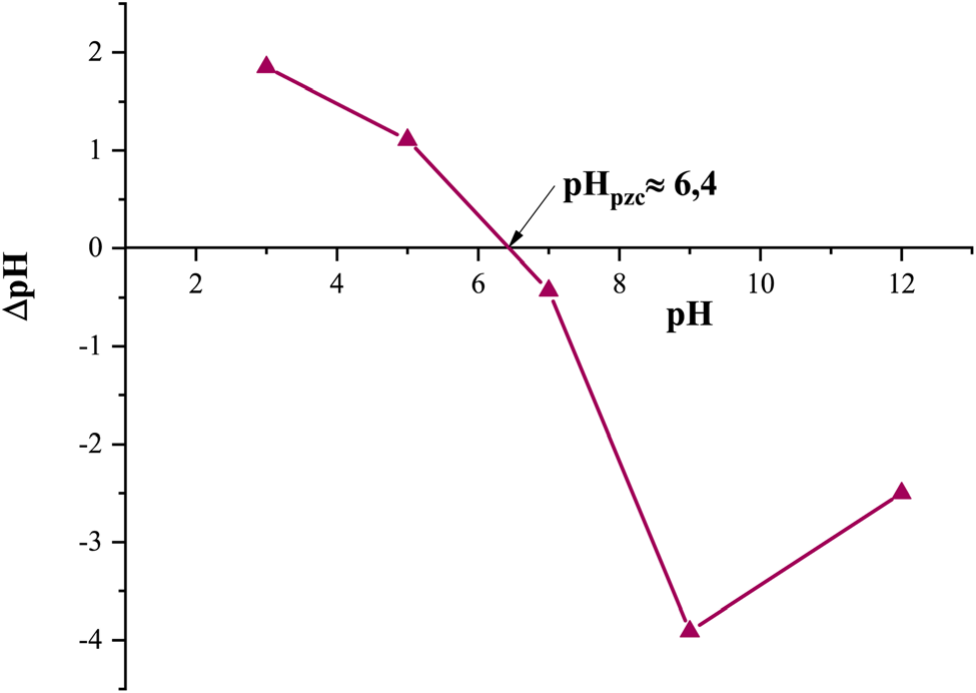
Point of zero charge (pH_pzc_) of the Fe_0.5_-HAp_Bio_ catalyst.

The ΔpH variation as a function of initial pH (Fig. 8) shows that the pH_pzc_ of the Fe_0.5_-HAp_Bio_ catalyst is around 6.4. Below this value, the catalyst surface is positively charged, while above this pH, it becomes negatively charged.

Furthermore, the initial pH of the CFX-Na solution is also 6.4. At this pH, the catalyst surface is practically neutral, which significantly limits the interactions between CFX-Na and Fe_0.5_-HAp_Bio_. This explains the low adsorption capacity observed experimentally (Fig. 7(b)).

All of these results demonstrate that the CFX-Na removal in this system is based primarily on catalytic reactions rather than on the pollutant adsorption onto the material.

##### 3.2.2.2. Catalyst behavior in aqueous solution

###### 3.2.2.2.1 pH evolution

**Fig. 9:**
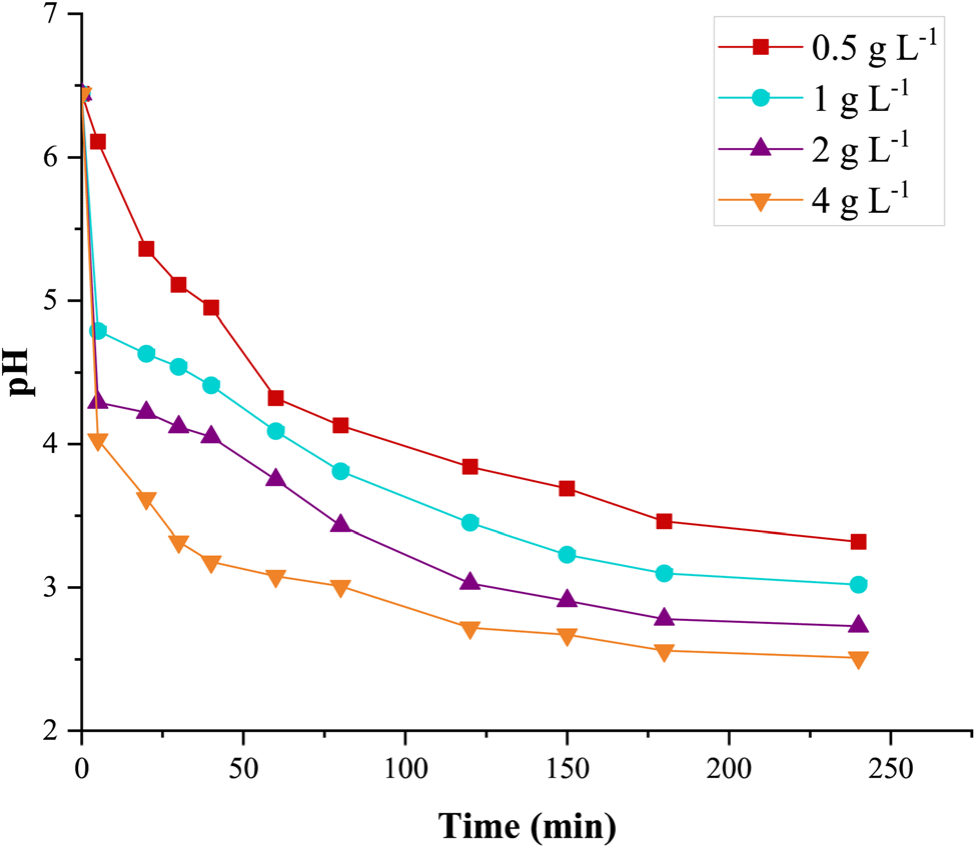
Effect of Fe_0.5_-HAp_Bio_ concentration (in suspension) on pH variation in a CFX-Na solution: [CFX-Na]_0_ = 0.15 mM, *I* = 400 mA, [Fe_0.5_-HAp_Bio_]_0_ = 0.5–4 g L^−1^.

The variation of the solution's pH compared to its initial value (around 6.4) was measured by varying the initial concentration of the Fe_0.5_-HAp_Bio_ suspension up to 4.0 g L^−1^. The results are presented in Fig. 9.

This figure shows that the pH of the solution progressively decreased from 6.44 to 5.36, 4.63, 4.22, and 3.62 within the first 20 minutes, as Fe_0.5_-HAp_Bio_ concentrations of 0.5, 1, 2, and 4 g L^−1^ were added, respectively.

The pH continued to decrease gradually, reaching more acidic values between 3.01 and 2.51 at the end of the treatment, when the Fe_0.5_-HAp_Bio_ concentration increased from 1 to 4 g L^−1^. These results show that increasing the Fe_0.5_-HAp_Bio_ concentration promotes greater release of protons, which is contributing to the result in self-acidifying the solution.

Such acidification may be explained by the formation of hydroperoxyl radicals (˙O_2_H) generated through Fenton-like reactions (eqn (2) and (8)). Indeed, this radical ˙O_2_H dissociation to the superoxide anion (O_2_˙^−^), as shown in eqn (9),^49^ leads to further acidification due to the increase in protons concentration, which directly affects the pH variation during electrolysis.

8 ≡Fe^III^ + H_2_O_2_ → ≡Fe^II^ + H^+^ + ˙O_2_H

9 ˙O_2_H ↔ O_2_˙^−^ + H^+^

10 –OH_surf_ ↔ H^+^ + =O_surf_ + e^−^

Also, the presence of hydroxyl functional groups OH^−^ on the hydroxyapatite surface can be considered as the cause of this acidification (eqn (10)). These groups are able to supply protons, which facilitates the transport of Fe^3+^ to the cathode during the treatment process.^50^

###### 3.2.2.2.2 Dissolved iron concentration during the electrolysis

**Fig. 10:**
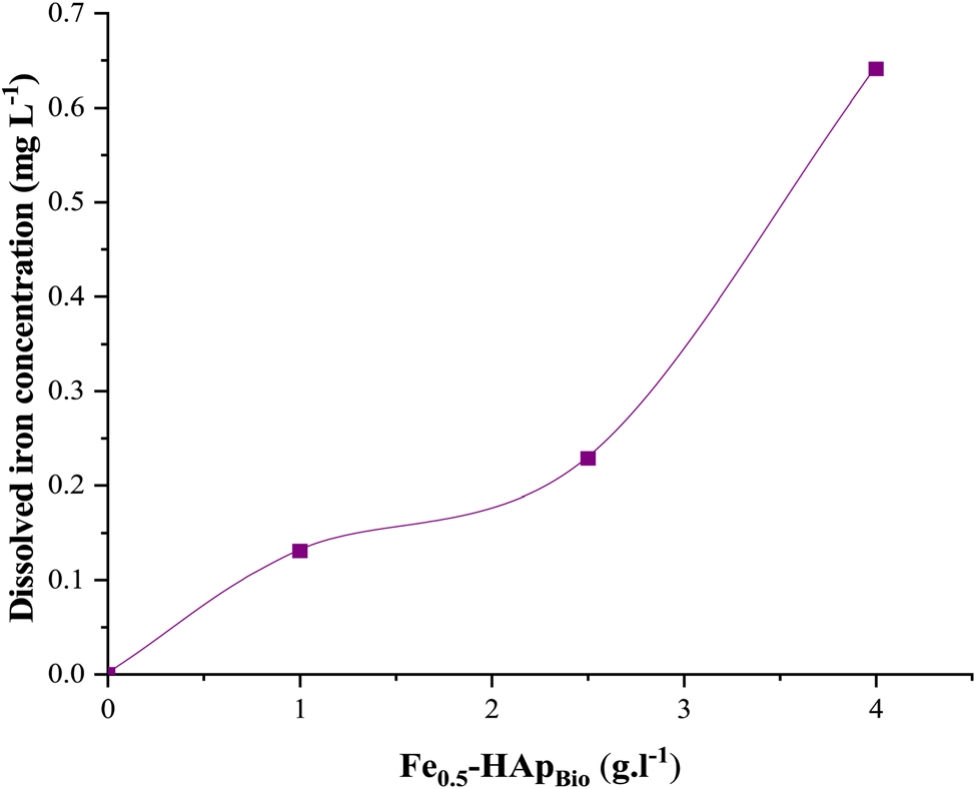
Effect of Fe_0.5_-HAp_Bio_ concentration (in suspension) on the variation of dissolved iron concentration in solution during an 80 min electrolysis: [CFX-Na]_0_ = 0.15 mM, *I* = 400 mA, [Fe_0.5_-HAp_Bio_]_0_ = 0.5, 1, 2.5 and 4 g L^−1^.

In heterogeneous electro-Fenton process, reactions predominantly take place at the catalyst surface and only limited quantities of dissolved iron ions are leached into solution, hence, leading to the radical's generation.^43^ Measuring the concentration of dissolved iron ions is therefore a crucial measure of catalytic activity, material stability, and the catalyst's contribution towards the mechanism of reactions. Based on this, ICP-OES was used to monitor the release of iron ions of Fe_0.5_-HAp_Bio_ at an initial pH of 6.4 during electrolysis, and the obtained results are shown in Fig. 10.

This figure shows that the concentration of dissolved iron (Fe^2+^/Fe^3+^) increases from 0.13 to 0.64 mg L^−1^ when the Fe_0.5_-HAp_Bio_ load increases from 1 g L^−1^ to 4 g L^−1^. These results demonstrate an almost linear correlation between dissolved iron and the amount of Fe_0.5_-HAp_Bio_. Based on the total iron content of the catalyst determined by XRF, the fraction actually leached remains very limited, less than 0.3%, even at high catalyst loads, demonstrating the stability of the catalyst. These observations confirm that the material can provide a controlled amount of dissolved iron ions under appropriate pH conditions, thereby enhancing the homogeneous Fenton reaction through a redox reaction with H_2_O_2_ and dissolved iron, which promotes further degradation of CFX-Na.

A similar behavior, combining both homogeneous and heterogeneous catalytic mechanisms, has been reported during EF treatment using different catalysts, such as Fe_3_O_4_ magnetite,^51^ iron supported on bentonite^52^ and iron supported on ion-exchange resin.^53^

Moreover, according to Moroccan quality standards for the reuse of treated wastewater for irrigation, the concentration of dissolved iron in this system remains well below the permissible limit of 5 mg L^−1^,^54,55^ meaning that secondary environmental contamination by this metal does not present a significant risk.

#### 3.2.3. Influence of experimental parameters on the degradation kinetics and mineralization of CFX-Na by heterogeneous electro-Fenton

##### 3.2.3.1. Effect of Fe_0.5_-HAp_Bio_ catalyst concentration

**Fig. 11:**
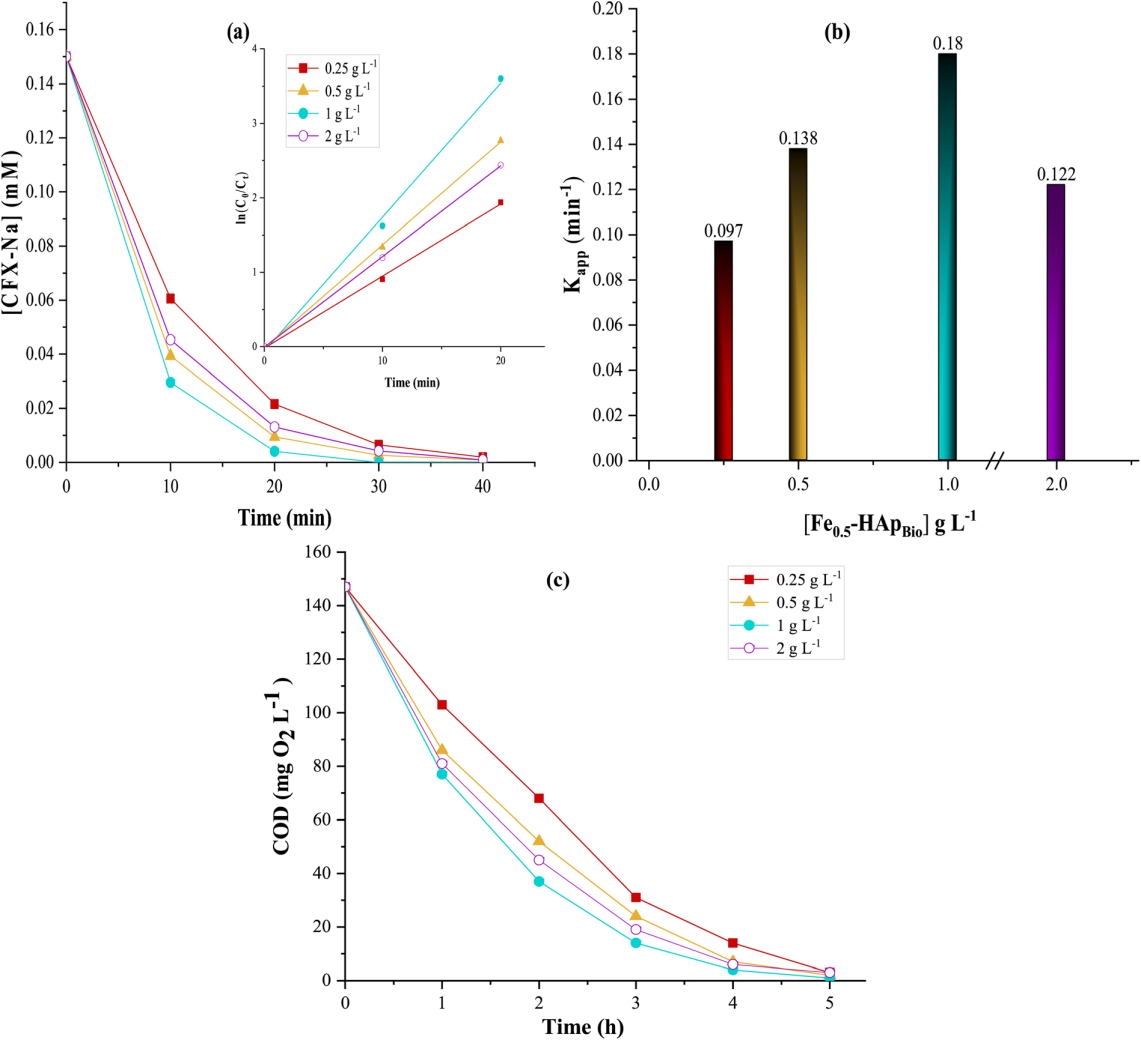
Effect of Fe_0.5_-HAp_Bio_ concentration on: (a) degradation kinetics, (b) apparent rate constants (*K*_app(CFX-Na)_), and (c) COD evolution; for CFX-Na oxidation by heterogeneous EF at initial solution pH: [CFX-Na]_0_ = 0.15 mM, *I* = 400 mA.

It is important to note that catalyst concentration plays a crucial role in the efficiency of the heterogeneous EF process, as it controls the generation of reactive species through the Fenton reaction.^56^ The influence of Fe_0.5_-HAp_Bio_ catalyst concentration on the degradation and mineralization efficiency of CFX-Na was investigated by varying the catalyst concentration from 0.25 to 2 g L^−1^, under an applied current of 400 mA. The results are illustrated in Fig. 11.

As expected, the degradation efficiency of CFX-Na was enhanced with increasing catalysts dosage (Fig. 11(a)). In fact, 98% degradation rate was obtained by the addition of 1 g L^−1^ catalyst after 20 minutes of electrolysis, while 85% was obtained with 0.25 g L^−1^ of catalyst. This phenomenon can be explained by the fact that increasing the catalyst increases available active sites on the catalyst surface, which facilitates the decomposition of H_2_O_2_, subsequently leading to a significant increases in reactive species in the reaction medium, thus accelerating the CFX-Na degradation.^57^

In contrast, the low catalyst concentration results in a limited and slower generation of oxidizing radicals and low process efficiency. The kinetic analysis of the degradation curve (Fig. 11(b)) has confirmed these observations with an apparent rate constant (*K*_app_) increasing directly proportional to the Fe_0.5_-HAp_Bio_ concentration to a maximum value of 0.18 min^−1^ at 1 g L^−1^, thus confirming the positive effect of the increasing number of active sites on the degradation efficiency.

However, at higher catalysts concentrations (*i.e.*, 2 g L^−1^), the degradation rate of CFX-Na reduced slightly with a lower apparent constant of 0.122 min^−1^. This phenomenon can be explained by the particle agglomeration of excess Fe_0.5_-HAp_Bio_, resulting in a loss of its active surface area, and by the scavenging of reactive species, especially ˙OH and ˙O_2_H radicals, by iron species, leading to undesirable reactions (eqn (11)–(13)).^58^ In addition, an excessive amount of ˙OH radicals may be consumed through self-recombination reactions (eqn (14) and (15)).^59^

11 Fe^III^ + ˙O_2_H → Fe^II^ + O_2_ + H^+^

12 Fe^II^ + ˙OH → Fe^III^ + OH^−^

13 Fe^II^ + ˙O_2_H → Fe^III^ + O_2_H^−^

14 ˙O_2_H + ˙OH → O_2_ + H_2_O

15 ˙OH + ˙OH → H_2_O_2_

The same tendency was observed for mineralization (Fig. 11(c)). In fact, increasing the Fe_0.5_-HAp_Bio_ concentration from 0.25 to 1 g L^−1^ resulted in an increase in mineralization efficiency from 90 to 98% after 4 hours of electrolysis. However, excessive catalyst concentration loading negatively affects treatment efficiency, probably due to the apparent increase in parasitic reactions, which consume the oxidizing radicals. Based on these results, the optimal catalyst concentration of 1 g L^−1^ was chosen for further experiments, which implies high mineralization efficiency and reasonable catalysts cost.

##### 3.2.3.2. Effect of the applied current

**Fig. 12:**
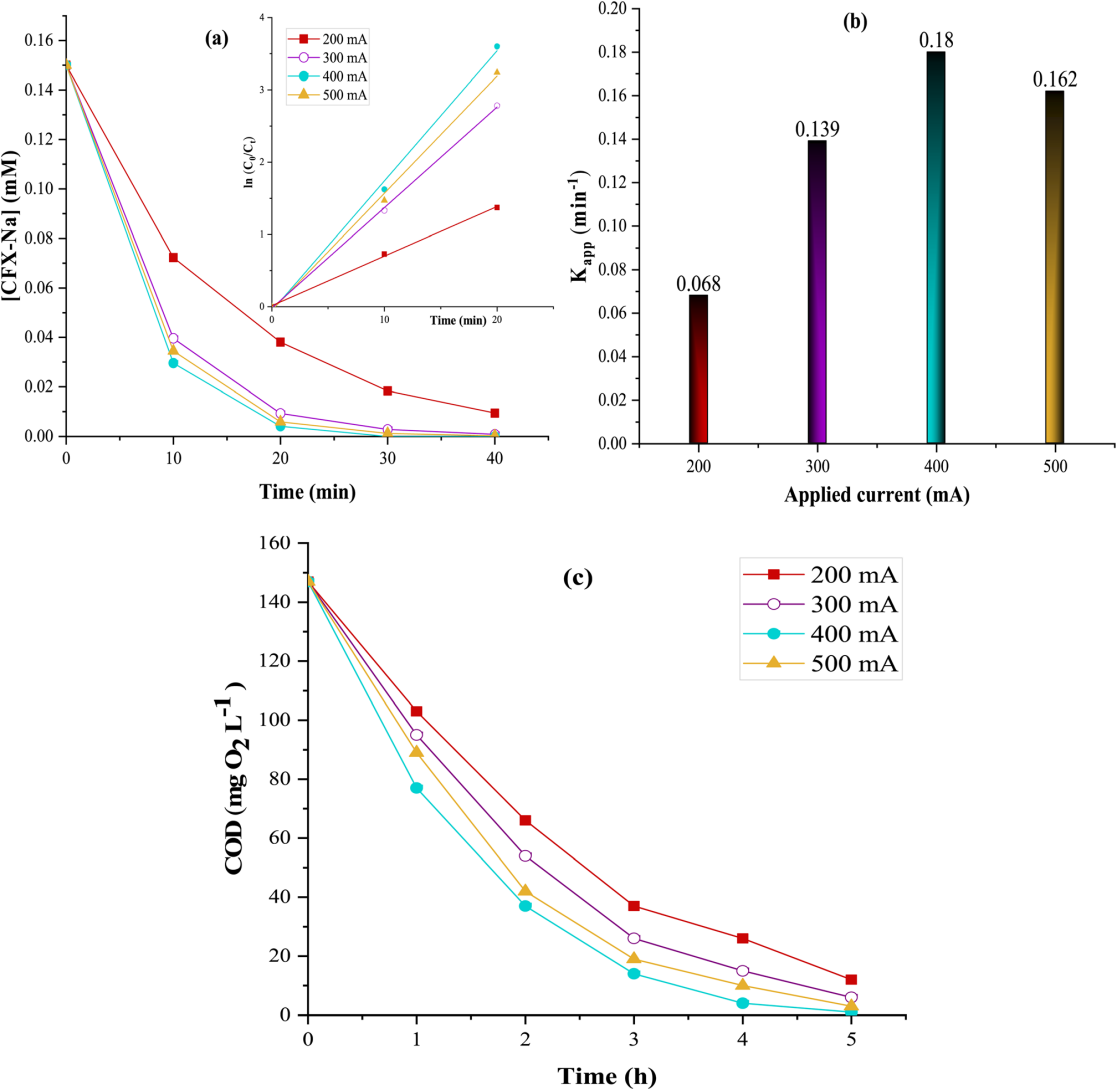
Applied current effect on: (a) degradation kinetics, (b) apparent rate constants (*K*_app(CFX-Na)_), and (c) COD evolution, during CFX-Na treatment by heterogeneous EF: [CFX-Na]_0_ = 0.15 mM, [Fe_0.5_-HAp_Bio_]_0_ = 1 g L^−1^.

The applied current is the most critical parameter influencing the efficiency and performance of electrochemical processes, as it controls the rate of H_2_O_2_ production, catalyst regeneration rate, and, consequently, the formation rate of reactive species *via* the Fenton reaction (eqn (1)).^60,61^ To evaluate its effect on the degradation and mineralization of CFX-Na in aqueous medium, a series of experiments was carried out in the presence of 1 g L^−1^ of Fe_0.5_-HAp_Bio_, while varying the applied current from 200 to 500 mA. All the results obtained are presented in Fig. 12.

These results clearly show that the degradation kinetics of CFX-Na significantly increase with the applied current rising from 200 to 400 mA (Fig. 12(a)). Indeed, after 25 min of electrolysis, the CFX-Na degradation efficiency rose from 77% to complete removal when the current intensities were increased from 200 to 400 mA. This behavior can mainly be attributed to:^62^

(i) Accelerated electro-regeneration of the ≡Fe^II^ catalyst at higher currents due to the cathodic reduction of ≡Fe^III^;

(ii) Enhanced anodic water oxidation with increasing applied current, creating synergy with catalyst regeneration at the cathode, thereby improving degradation of organic matter in water;

(iii) *In situ* electro-generation of H_2_O_2_ by cathodic oxygen reduction (eqn (3)), which is proportionally dependent on the applied current.

This is also confirmed by the apparent rate constant (*K*_app_) determined during CFX-Na oxidation (Fig. 12(b)), which increased from 0.068 to 0.18 min^−1^ as the current increased from 200 to 400 mA.

However, when the current was further increased to 500 mA, no significant improvement was observed in the degradation profile, and *K*_app_ slightly decreased to 0.162 min^−1^. This phenomenon could probably be attributed to the increase in side reactions, in particular the O_2_ evolution at the anode surface (eqn (16)), which slows down the anodic oxidation rate, as well as H_2_ gas evolution at the cathode (eqn (17)).^63^

16 2H_2_O → O_2_ + 4H^+^ + 4e^−^

17 2H_2_O + 2e^−^ → H_2_ + 2OH^−^

A similar trend was observed when continuing the treatment toward total mineralized of solution into CO_2_, H_2_O, and inorganic ions (Fig. 12(c)). In fact, a significant increase in current implies a progressive decrease in COD, reaching 93% COD removal after 4 hours of electrolysis at 500 mA, compared to 98% at 400 mA. This is due to various competing reactions, such as oxygen and hydrogen evolution at the anode and the cathode, respectively (eqn (16) and (17)).^63^

#### 3.2.4. Catalytic oxidation mechanism by the Fe_0.5_-HAp_Bio_ catalyst

The heterogeneous reaction is a surface-controlled process that mainly depends on the H_2_O_2_ concentration, the properties of the supported catalyst, and the pH of the solution. It is commonly accepted that the generation of oxidizing radicals from the H_2_O_2_ decomposition catalyzed by supported iron entities is the critical step in the overall oxidation process, which corresponds to the Haber–Weiss cycle mechanism, similarly to the classical homogeneous EF reaction.^56^ Furthermore, it is well known that ˙OH radicals are the major contributors to the electrochemical degradation of contaminants, due to their high reactivity and non-selectivity. However, the reaction between Fe(iii) species and H_2_O_2_ mainly produces ˙O_2_H radicals.

Therefore, it is important to clarify the contribution of ˙OH and ˙O_2_H/O_2_˙^−^ reactive species during the electrochemical degradation of CFX-Na in the presence of Fe_0.5_-HAp_Bio_ as a heterogeneous catalyst, in order to better understand the role of these radicals and the possible reaction pathways in the heterogeneous phase.

**Fig. 13:**
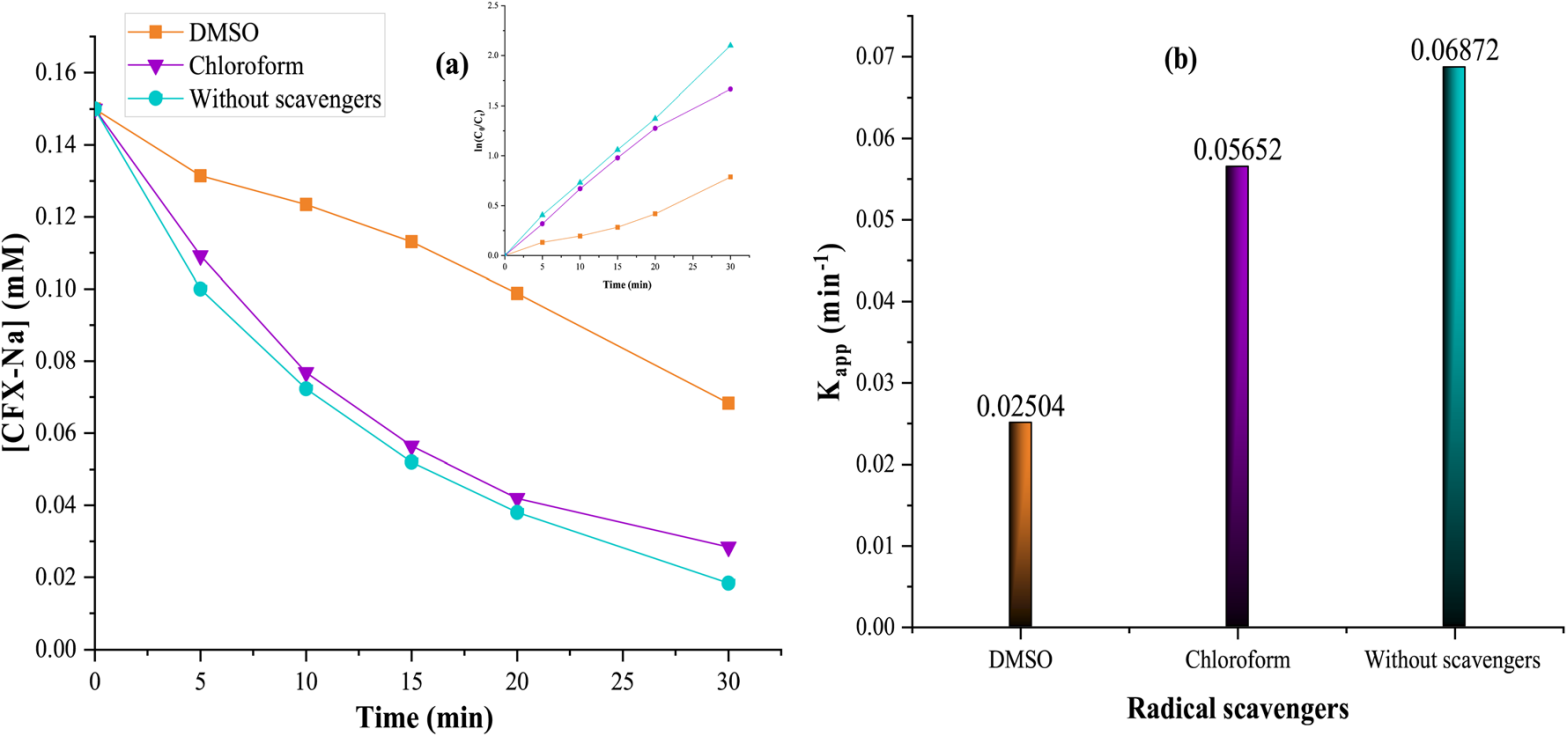
Electrochemical degradation of CFX-Na by heterogeneous EF process in the absence and presence of DMSO and chloroform. (a) Degradation kinetics, including the inset graph corresponding to ln(*C*_0_/*C*_t_) as a function of time, (b) apparent rate constants (*K*_app_ (CFX-Na)): [CFX-Na]_0_ = 0.15 mM, [Fe_0.5_-HAp_Bio_] = 1 g L^−1^, [DMSO] = [chloroform]= 0.25 M, *I* = 100 mA.

In this regard, radical scavenging tests were performed using dimethyl sulfoxide (DMSO) and chloroform as radical scavengers. DMSO was selected for its high reactivity towards hydroxyl radicals ˙OH, with a rate constant of 6.6 × 10^9^ M^−1^ s^−1^. Its oxidation in the medium leads to the formation of methanesulfonic acid and methyl radical (eqn (18)).^64,65^18
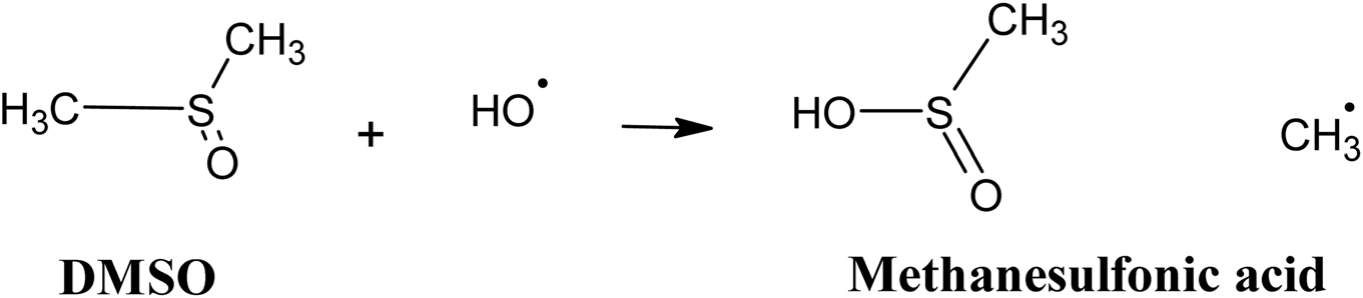
In addition, chloroform was used as a superoxide radicals O_2_˙^−^ scavenger. This compound shows strong reactivity with O_2_˙^−^, with a rate constant of 9.6 × 10^8^ M^−1^ s^−1^, while its reactivity with the ˙OH radical is relatively low (rate constant of 7 × 10^6^ M^−1^ s^−1^).^66^ The results obtained in the absence and presence of these scavengers are illustrated in Fig. 13.

The results indicate that 89% degradation of CFX-Na was achieved after 30 min of treatment in the absence of scavengers. However, the addition of DMSO significantly inhibited the degradation, reducing it to 54%. This inhibition was also reflected in a decrease in the rate constant, from 0.06872 min^−1^ without DMSO to 0.02504 min^−1^ in its presence.

Similarly, the introduction of chloroform also slowed the degradation rate, through less significantly than with DMSO. These results confirm the major involvement of ˙OH radicals in the electrochemical oxidation reaction mediated by Fe_0.5_-HAP_Bio_, and that the degradation mechanism proceeds efficiently in the presence of this radical, while suggesting that ˙O_2_H/O_2_˙^−^ radicals play a secondary role during electrolysis in the CFX-Na degradation.

**Fig. 14:**
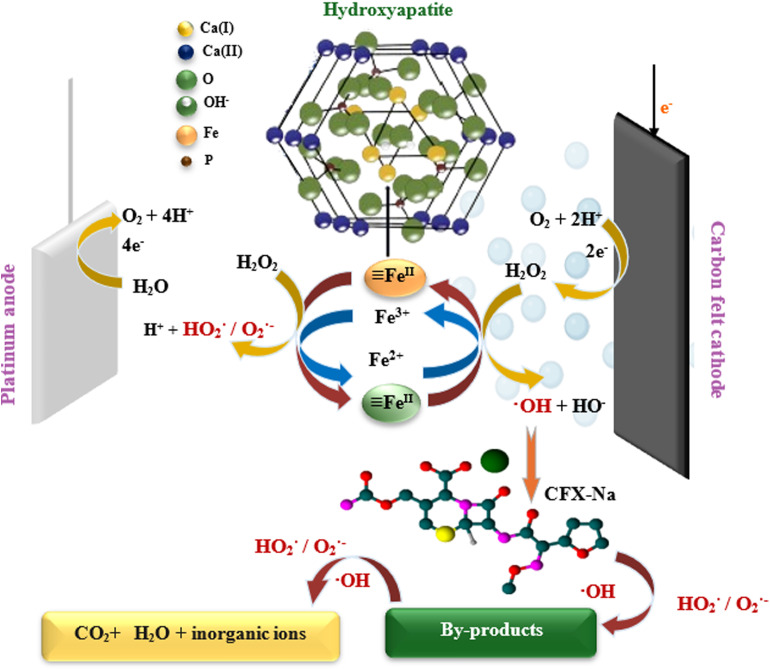
Schematic representation of the oxidation mechanism *via* the heterogeneous EF process using the Fe_0.5_-HAp_Bio_ catalyst.

Based on the above results, a reaction mechanism for the heterogeneous electro-Fenton process using Fe_0.5_-HAp_Bio_ particles was proposed (Fig. 14).

First, H_2_O_2_ was efficiently electro-generated by the reduction of dissolved oxygen O_2_ at the cathode (eqn (3)). This reaction is favored in an acidic medium, as shown by the pH evolution (Fig. 8), where a decrease in pH was observed. Indeed, an acidic medium ensures a sufficient concentration of protons necessary for this reaction. Once generated *in situ*, H_2_O_2_ is activated on the surface of the iron-containing solid catalyst.

In our case, where ferric active sites are already available on the Fe_0.5_-HAP_Bio_ catalyst surface, hydrogen peroxide reacts with these ferric sites, leading to their conversion into ferrous species (eqn (19)).

19 HAp ≡ Fe^III^ + H_2_O_2_ → HAp ≡ Fe^II^ + ˙O_2_H + H^+^

The formed hydroperoxyl radical (˙O_2_H) may dissociate into a superoxide anion (˙O_2_^−^) and a hydrogen ion (H^+^), as previously mentioned in eqn (9). In addition, the ˙O_2_H and O_2_˙^−^ radicals play an important role in the iron redox cycle, as they can react with ferric sites to regenerate ferrous active sites (eqn (20) and (21)).

20 HAp ≡ Fe^III^ + ˙O_2_H → HAp ≡ Fe^II^ + O_2_ + H^+^

21 HAp ≡ Fe^III^ + O_2_˙^−^ → HAp ≡ Fe^II^ + O_2_

The Fe^II^ formed on the catalyst surface reacts with H_2_O_2_ to generates hydroxyl radicals ˙OH, according to the classic Fenton (eqn (22)). Moreover, the increase in protons concentration promotes the dissociation of H_2_O_2_ into ˙OH, as shown in eqn (23),^67^ further confirming the main role of these radicals in activating the catalytic system.

22 HAp ≡ Fe^II^ + H_2_O_2_ → HAp ≡ Fe^III^ + ˙OH + OH^−^

23 H_2_O_2_ + e^−^ + H^+^ → ˙OH + H_2_O

In addition, the portion of iron dissolved into the reaction medium (Fig. 9) also catalyzes the decomposition of H_2_O_2_ to generate oxidizing radicals, *via* a Haber–Weiss mechanism, where dissolved iron ions and surface Fe^II^/Fe^III^ sites (eqn (24)) immediately react with H_2_O_2_. Furthermore, the main regeneration of Fe^II^/Fe^2+^ ions would be achieved through a direct cathodic electro-reduction of Fe^II^/Fe^3+^ ions (eqn (24) and (4)). Finally, upon attack by the generated radicals, the antibiotic CFX-Na can be mineralized into H_2_O, CO_2_, and inorganic ions (eqn (5)).

24 ≡Fe^III^ + 1e^−^ → ≡Fe^II^

#### 3.2.5. Catalyst recycling and stability

Generally, catalyst lifetime is a crucial factor in the economic viability of a process, as it depends on chemical, thermal and mechanical stability. This stability can be influenced by many factors, among which are decomposition, combustion, and contamination;^68^ and is necessary to ensure efficient catalysts reuse. Indeed, reusability is one of the major benefits of heterogeneous catalysts compared to their homogeneous counterparts.^69^

In practice, the catalyst should be easily separated from the treated effluent and reused several times without any significant reduction in activity. Therefore, ensuring the long-term stability of the catalyst is essential to minimize the cost of operations and to achieve a consistent effluents quality, thereby ensuring the optimum and sustainable treatment performance.

**Fig. 15:**
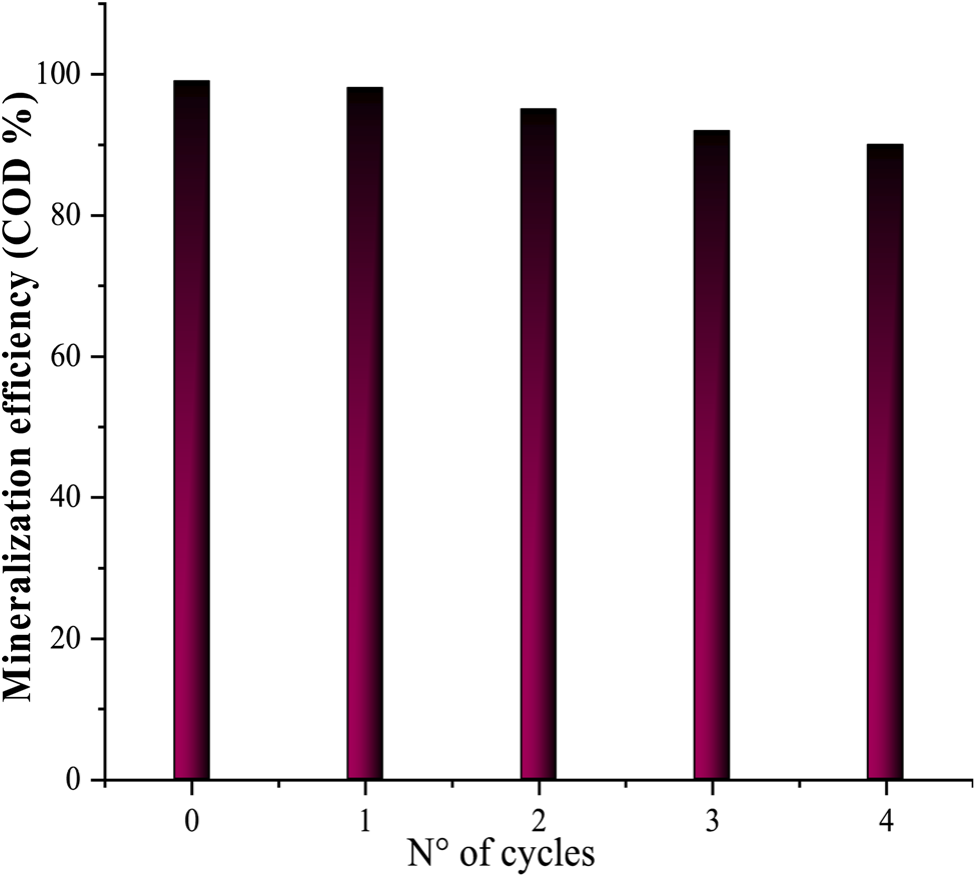
Reuse of the Fe_0.5_-HAp_Bio_ catalyst for CFX-Na mineralization at the initial solution pH: [CFX-Na] = 0.15 mM, *I* = 400 mA, [Fe_0.5_-HAp_Bio_]_0_ = 1 g L^−1^.

Reusability of the iron-doped biomaterial (Fe_0.5_-HAp_Bio_) was measured by conducting successive electrolysis for CFX-Na mineralization at the same initial pH conditions. Five reuse cycles were conducted under the same conditions as the first cycle. The catalyst was separated after each test by simple filtration followed by several rinses using the double-distilled water and dried at 80 °C and used in the next cycle. The results obtained for the five cycles are shown in Fig. 15.

As shown in the figure, after five successive reuse cycles, the catalytic efficiency of the material remained high during the 5 hours electrolysis periods. Over 92% of CFX-Na was removed after the fifth cycle, compared to 98% in the first cycle.

This result indicates that the Fe_0.5_-HAp_Bio_ exhibits excellent reusability and good stability at initial pH under repeated operating conditions, also suggesting that the loss of active sites on the catalyst surface during the heterogeneous EF process is negligible.

#### 3.2.6. Energy consumption

**Fig. 16:**
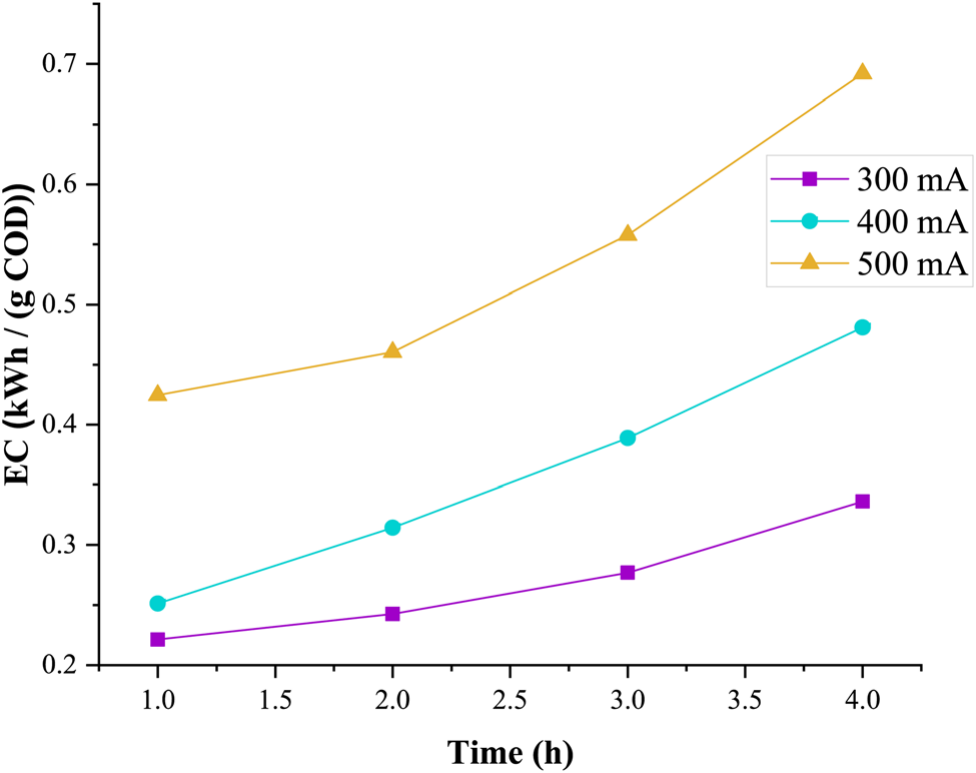
Energy consumption (kWh per g COD) at different current intensities during the heterogeneous EF process using the biocatalysts Fe_0.5_-HAp_Bio_: [CFX-Na] = 0.15 mM, [Fe_0.5_-HAp_Bio_]_0_ = 1 g L^−1^.

Energy consumption was evaluated as a function of treatment duration and applied current intensity during the mineralization of a 0.15 mM CFX-Na solution *via* the heterogeneous EF process using Fe_0.5_-HAp_Bio_, based on eqn (7). The results, expressed in kWh per g of COD removed (kWh per g(COD)), are illustrated in Fig. 16.

The figure shows that at higher current intensities and especially with longer treatment durations (up to 4 hours), energy consumption significantly increased, specifically, after a 1 hour of treatment at 300 mA, energy consumption was approximately 0.2212 kWh per g COD, while increasing the current to 500 mA led to a substantial rise in consumption, reaching around 0.4246 kWh per g COD. Thus, by increasing the duration of electrolysis, the electrical energy consumption (in kWh) also increased for each g of COD removed.

This phenomenon can be explained by the mineralization behavior observed in Fig. 12(c), which indicated a decrease in the mineralization rate over time. This deceleration may be explained by the gradual decrease of available organic matter and by the participation of reactive radicals in parasitic reactions, which leads to an increase energy consumption. Additionally, the low reactivity of radicals toward the formed by-products also contributes to the rise in energy consumption.

### 3.3. Hybrid process: bio-heterogeneous electro-Fenton

#### 3.3.1. Biodegradability assessment

Improving the biodegradability of treated solutions is a key requirement for ensuring the effective performance of biological post-treatment, allowing not only determines whether a solution is likely to be biological degradation, but also helps estimate the duration required to implement the hybrid process. To this end, biodegradability tests were carried out on 0.15 mM CFX-Na solutions treated with Fe_0.5_-HAp_Bio_ under an optimal applied current of 400 mA.

**Fig. 17:**
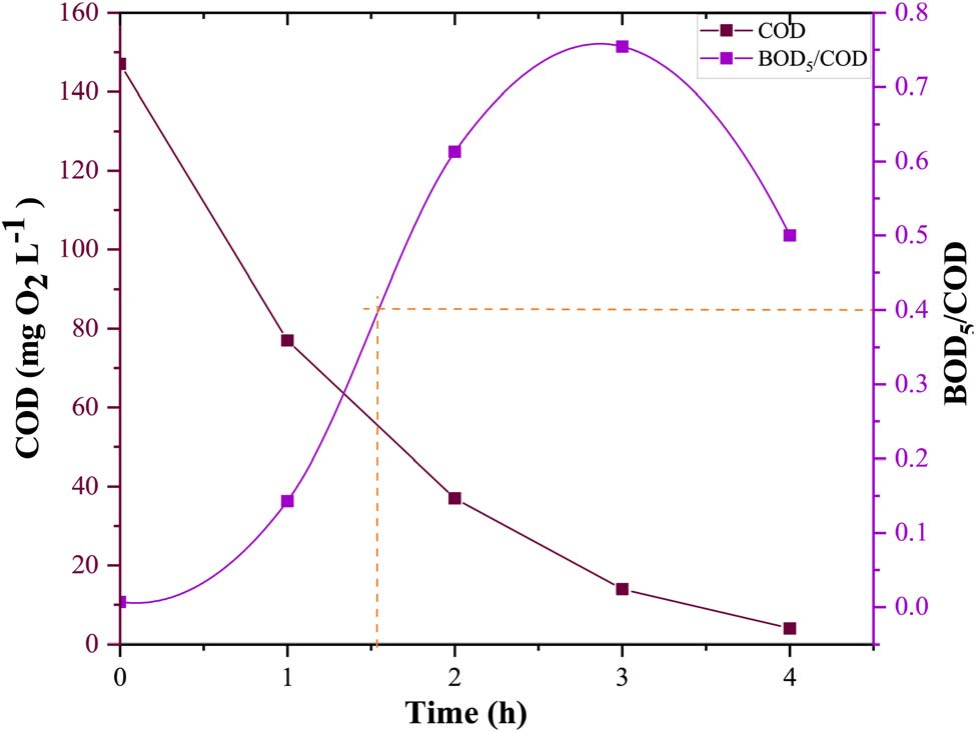
Biodegradability evolution (BOD_5_/COD ratio) of solutions treated by heterogeneous EF: [CFX-Na]_0_ = 0.15 mM, *I* = 400 mA, [Fe_0.5_-HAp_Bio_]_0_ = 1 g L^−1^.

The results (Fig. 17) show that the BOD_5_/COD ratio, a commonly used biodegradability indicator, gradually increases, reaching a maximum of approximately 0.7 after 2 hours of treatment. Nevertheless, a value of 0.4 for the BOD_5_/COD ratio, which is widely considered the threshold above which a solution is regarded as biodegradable,^38,70^ was already achieved after only 90 minutes of heterogeneous EF electrolysis.

This improvement can be attributed to the breakdown of initially refractory compounds, into short-chain intermediates, which are more easily assimilated by microorganisms, making the solution suitable for low-cost biological treatment.

#### 3.3.2. Aerobic biological treatment using activated sludge

The enhanced biodegradability of the contaminated solution after electrolysis suggests that during heterogeneous electrochemical treatment using Fe_0.5_-HAp_Bio_, CFX-Na oxidizes into simpler compounds that are more easily assimilated by microorganisms.

**Fig. 18:**
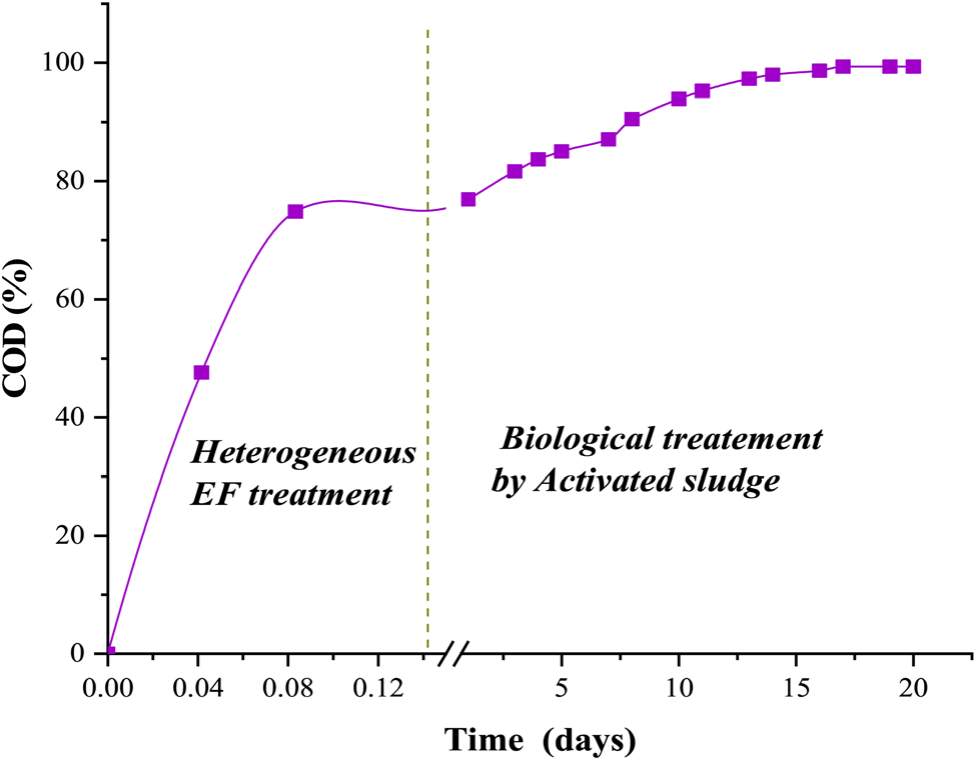
CFX-Na mineralization rate during biological treatment using activated sludge at 25 °C, pH = 7 of a CFX-Na solution pretreated for 90 min: [CFX-Na]_0_ = 0.15 mM, *I* = 400 mA, [Fe_0.5_-HAp_Bio_]_0_ = 1 g L^−1^.

For this purpose, a CFX-Na solution pretreated for 90 minutes *via* the heterogeneous EF process was subjected to aerobic treatment using activated sludge, conducted in duplicate over a 21 day period, aiming to biologically degrade the remaining by-products. The efficiency of the hybrid process was examined by monitoring the evolution of mineralization throughout the biological treatment, and the results obtained are presented in Fig. 18.

This figure shows that the COD removal rate was higher during the initial stage of the biological treatment. In particular, the mineralization rate of the CFX-Na by-products generated during the pretreatment process grew faster, attaining 85% within the first 5 days of cultivation. This rapid reduction can be attributed to the biodegradability of certain degradation products, and therefore they are more easily assimilated by the microorganisms.^71^ On the fifth day, COD removal continued to increase albeit at a slower rate.

This trend indicates that the biological process is an effective complement the degradation initiated by heterogeneous EF treatment, reaching 99% mineralization after 17 days. These results highlight the potential of the hybrid aerobic bio-heterogeneous EF process as an efficient and viable method for treating wastewater contaminated with pharmaceutical residues, as it can combine the rapidity performance of heterogeneous electrochemical oxidation with the sustainability of biological treatment, which guarantees almost total decontamination of pollutants and significantly reduced treatment costs.

## 4. Conclusion

This paper notes the potential of a heterogeneous catalyst based on iron-doped hydroxyapatite derived from bovine bone (Fe_0.5_-HAp_Bio_), for the electrochemical treatment of CFX-Na through electro-Fenton process. Structural characterization confirmed that the crystalline structure of HAp_Bio_ remains unchanged by the incorporation of iron, and the IR analysis revealed the presence of functional groups of hydroxyls OH^−^, water H_2_O, phosphate PO_4_^3−^ and carbonate CO_3_^2−^ ions.

Iron dispersion was also found to be homogeneous, which maximizes the availability of active sites and improves the global catalytic performance. Catalytic tests showed that optimum doping with 0.5% iron allowed complete degradation of CFX-Na in 25 minutes with a kinetic constant of 0.18 min^−1^ and almost complete mineralization after 5 hours of electrolysis with a catalyst dose of 1 g L^−1^ and an applied current of 400 mA. In addition, the radical scavenging tests proved that hydroxyl radicals ˙OH were the dominant reactive species, with additional contributions from hydroperoxyl ˙O_2_H and superoxide O_2_˙^−^ radicals. The reaction mechanism was suggested, which involved the formation of reactive species through the reduction of H_2_O_2_ by the active sites on the catalyst surface, as well as by Fenton-like reaction involving dissolved iron in solution. Catalyst reuse tests demonstrated the good stability of catalyst during a series of treatment cycles.

In parallel, the biodegradability analysis (BOD_5_/COD ratio) indicated that the by-products produced were more biologically assimilable after 90 minutes of treatment. Lastly, combining the heterogeneous electro-Fenton process with aerobic biological treatment led to almost total mineralization (99%) during 17 days of incubations, reducing the consumption of energy costs, and enhancing the sustainability of process.

These results highlight the effectiveness and environmental relevance of iron-doped bio-hydroxyapatite as a catalyst, opening promising prospects for the valorisation of biological waste as a catalytic support in pharmaceutical wastewater treatment processes.

## Conflicts of interest

The authors have declared no conflicts of interest.

## Data Availability

All data generated or analysed during this study are included in this published article.
